# Photosystem-Based Biophotoelectrodes for Solar Energy Conversion

**DOI:** 10.1021/acs.chemrev.6c00021

**Published:** 2026-07-21

**Authors:** Huijie Zhang, Nicolas Plumeré

**Affiliations:** † School of New Energy, 12436 Nanjing University of Science and Technology, Jiangyin, Jiangsu 214443, China; ‡ 9184 Technical University of Munich, Campus Straubing for Biotechnology and Sustainability, Professorship for Electrobiotechnology, Uferstraße 53, 94315, Straubing, Germany

## Abstract

Photosystem-modified electrodes have been developed as alternatives to classical semiconductor-based systems for solar energy conversion into chemicals or electricity. Photosystems, the photoactive proteins of natural photosynthesis, offer exceptional light-harvesting efficiency and large charge separation and are composed entirely of earth-abundant elements. Their main limitations remain their limited robustness and difficulty of efficiently interfacing them with electrodes. Here, we review the strategies for integrating the three major classes of photosystems (photosystem II, photosystem I, and bacterial reaction centers) into functional devices. We examine electrode architectures such as biophotoanodes and biophotocathodes as well as their assembly into biased and bias-free biophotoelectrochemical cells using various protein immobilization approaches aimed at maximizing photocurrent and energy efficiency. Particular emphasis is placed on understanding electron-transfer pathways between electrodes and photosystems, including potential short-circuiting pathways. Finally, we highlight key characterization tools and performance metrics that are essential for identifying bottlenecks and guiding optimization.

## Graphical abstract

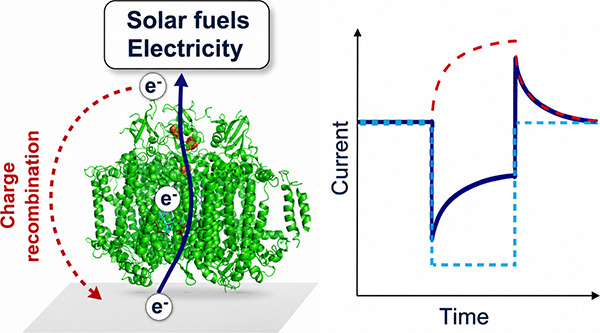

## 1 Introduction

The increasing global energy demand, coupled with the urgent need to mitigate climate change, has accelerated the search for sustainable and carbon-neutral energy sources. Among the renewable resources, solar energy is central, as it represents the most abundant and pervasive energy flux on earth.^1^ Harnessing even 0.01% of this energy holds the potential to satisfy humanity’s energy requirements.^2,3^ Conventional technologies for solar energy conversion, primarily solid-state photovoltaics, have seen remarkable advancement and deployment.^4–7^ Artificial photosynthesis systems, which aim to mimic nature to produce solar fuels (e.g., hydrogen, formate, or methanol), are also the subject of intense research.^8–13^ The main challenges in these areas lie in the development of light energy converting systems that combine the requirements of scalability, efficiency, robustness, and nontoxicity.^12,14^

Natural photosynthesis is one of the most fundamental processes that sustains life on earth and represents an inherently sustainable strategy for solar energy conversion. Through evolution, photosynthetic organisms have developed photosystems, which are highly efficient light-harvesting protein complexes specialized in converting absorbed photons into charge-separated states with quantum efficiencies approaching unity.^15–19^ Over the past two decades, an emerging subfield within solar energy research has focused on interfacing the biological photosynthetic machinery with artificial materials to create biohybrid systems for the light-driven generation of electrical or chemical energy.^15,20–22^ This Review concentrates on one of the most promising of these biohybrid strategies: photosystem-based biophotoelectrochemical cells, which aim to study and exploit the light-induced electron-transfer processes of photosynthetic proteins integrated into electrochemical cells.^16,23–25^

Biophotoelectrochemical cells can be constructed using any of the three major classes of natural photosynthetic protein complexes: photosystem I (PSI), photosystem II (PSII), or bacterial reaction centers (bRCs). A wide range of strategies for integrating photosystems with electrodes has led to steadily improving performance over the years, particularly in terms of achievable photocurrent densities (Figure 1). In these systems, light is absorbed directly by the photosynthetic protein, initiating charge separation and enabling the transfer of electrons either into an external electrical circuit for power generation or toward catalytic reactions for the production of solar fuels.^26,27^

This review is organized as outlined in Figure 2. We begin with an overview of the structure and function of photosystems within their native thylakoid membrane environment, followed by a discussion of the key criteria governing their selection, modification, and engineering for immobilization on electrode materials. We then describe the main strategies for integrating photosystems into biophotoelectrodes, emphasizing the central role of the biotic–abiotic interface in determining the performance and operational behavior of biophotoelectrochemical systems. Subsequently, we review the electrochemical and spectroscopic characterization methods used to identify bottlenecks in electron-transfer pathways and overall light-to-energy conversion, providing a framework for rational optimization of photosystem-modified electrodes. Finally, we highlight the broad range of applications enabled by these biohybrid assemblies, from direct electricity generation in biophotovoltaic devices to the production of sustainable solar fuels, with focus on the relevant performance metrics. By addressing these key aspects, this review aims to provide guidance for the future development of photosystem-based biophotoelectrochemical cells, summarizing both the substantial progress achieved to date and the remaining challenges on the path toward scalable and practical solar energy conversion.

**Figure 1:**
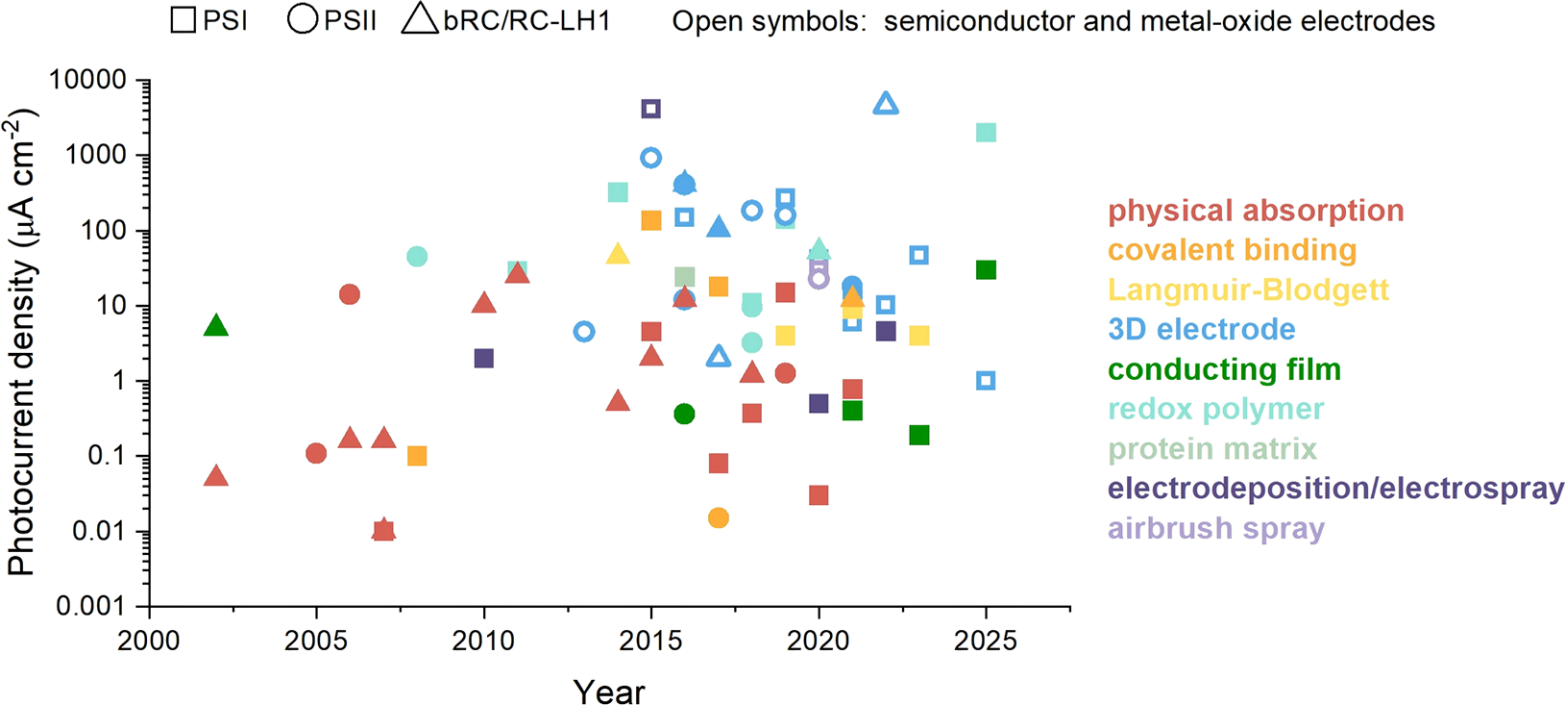
Progress in photocurrent density of the photosystem-based biophotoelectrodes in the past two decades for various electrode configurations.

## 2 Photosynthetic Proteins

Photosynthetic proteins, which initiate the photosynthetic electron transfer chain, feature high quantum efficiency in light-energy conversion (near 100%),^28^ fast intraprotein electron transfer rates,^29,30^ and large charge separation (1 V for PSI).^31^ These proteins vary across organisms within two types of photosynthesis, namely oxygenic photosynthesis and anoxygenic photosynthesis^32^ (Figure 3a,b).

### 2.1 Photosynthesis

Oxygenic photosynthesis, seen in plants, algae, and cyanobacteria, utilizes both PSI and PSII embedded in the thylakoid membrane.^33^ The overall photosynthetic electron transfer chain is described as the Z-scheme^34^ (Figure 3a). The process starts with PSII that oxidizes H_2_O and releases O_2_, protons, and electrons upon light absorption. The electrons are then transferred to PSI via an electron transport chain consisting of plastoquinone, the cytochrome *b*_6_ *f* complex (Cyt *b*_6_ *f*), and plastocyanin.^35,36^ A second light-induced charge separation at PSI provides the energy to ultimately transfer the electrons to ferredoxin and then to ferredoxin-NADP^+^ reductase (FNR) to reduce NADP^+^ to NADPH.^37^ Photosynthetic processes also pump protons into the thylakoid lumen through water oxidation and the redox cycling of plastoquinones. This generates a proton gradient that drives ATP synthesis by ATP synthase via chemiosmosis.^38^

Anoxygenic photosynthesis, found in organisms such as purple bacteria, green bacteria, and heliobacteria, relies on a single photosystem embedded in the cell membrane and uses electron donors such as hydrogen, hydrogen sulfide, or elemental sulfur instead of water; consequently, it does not evolve O_2_ ^39^ (Figure 3b). In *Rhodobacter (Rba.) sphaeroides*, photosynthesis is initiated when the bRC undergoes charge separation following excitation energy transfer from light-harvesting complexes.^40^ The resulting electrons are shuttled through a quinone pool to the cytochrome *bc*_1_ complex.^41^ There, oxidation of ubiquinol and reduction of cytochrome *c*_2_ are coupled to proton translocation across the membrane, generating a proton gradient that drives ATP synthesis.^42^ The reduced cytochrome *c*_2_ then diffuses back to the bRC, reducing its special pair and completing the cyclic electron-transfer pathway.^42^

Photosynthetic proteins are further classified according to the type of photosynthetic reaction center (RC) they contain, defined by the nature of the terminal electron acceptor: either a [4Fe-4S] cluster (type I, like PSI) or a quinone (type II, as in the purple bRC).^43^ PSII is the only enzyme capable of catalyzing the light-driven oxidation of water, while PSI functions as a highly efficient, though noncatalytic light-driven electron pump. The type II RC in purple bacteria is structurally and functionally analogous to PSII in that it uses quinone as the terminal electron acceptor; however, it lacks the water-oxidizing complex, rendering it substantially more stable than PSII. In this respect, it also resembles PSI in functioning primarily as a light-driven electron pump.

**Figure 2:**
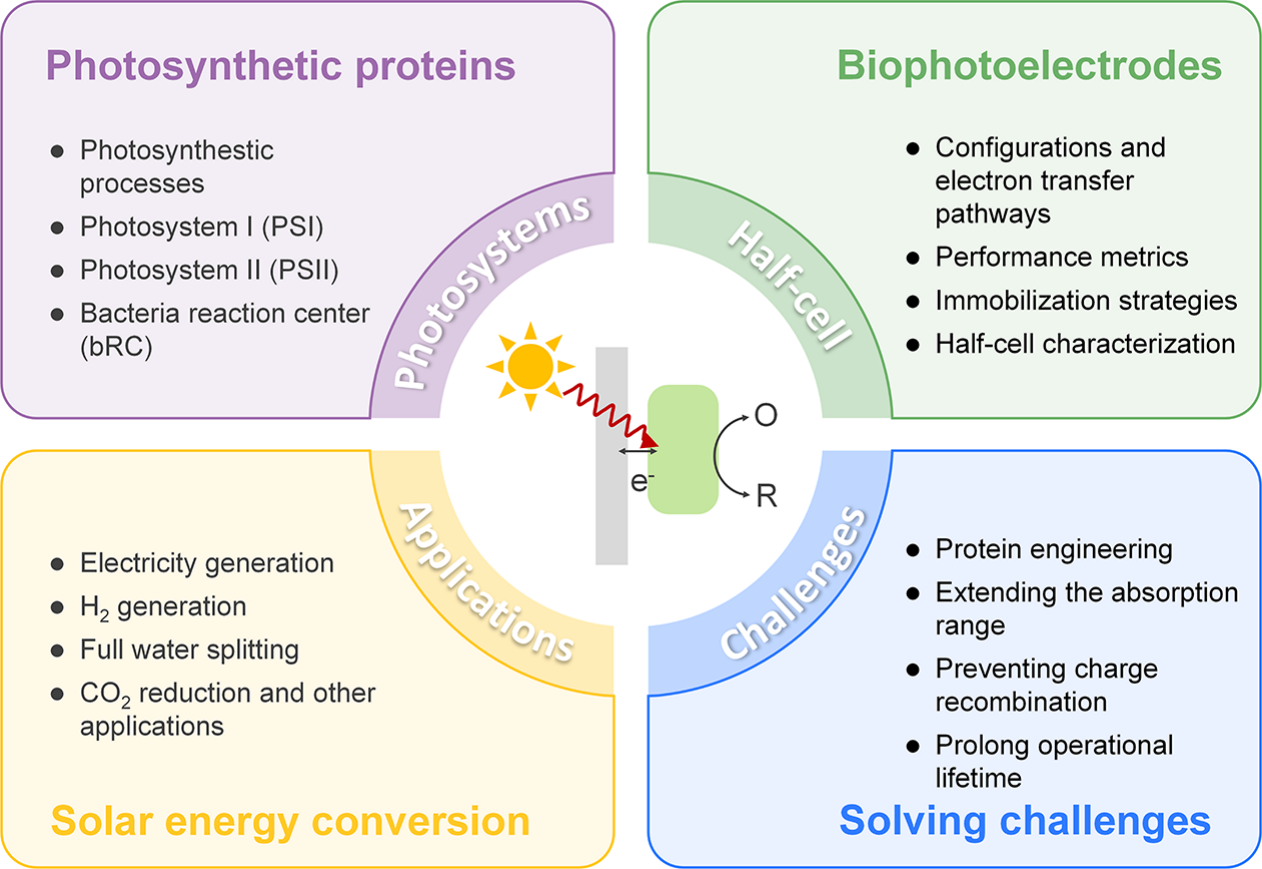
Overview of the content of this review.

### 2.2 PSII Structure and Function

PSII is a large multisubunit pigment–protein complex that initiates oxygenic photosynthesis. At its core lies a symmetric heterodimer composed of the D1 and D2 proteins, which provide the structural framework for the cofactors involved in charge separation and electron transport.^45–49^ This D1–D2 heterodimer is associated with two light-harvesting antenna subunits CP43 and CP47, which absorb photons and funnel excitation energy to the P680 RC composed of a special pair of chlorophyll *a* molecules within the D1–D2 core.^50^

Electron transfer is initiated when the primary electron donor P680 absorbs excitation energy delivered from the surrounding light-harvesting complexes (LHCII), promoting it to the excited state (P680*) (Figure 3c,f). This excited electron is rapidly transferred in a charge separation process, first to the electron acceptor pheophytin (Pheo) and then sequentially to two plastoquinone acceptors: first to the tightly bound Q_A_ and finally to the mobile Q_B_.^51,52^ After accepting two electrons as well as two protons from the stroma, Q_B_ is reduced to plastoquinol (PQH_2_), which diffuses away to transfer electrons further along the photosynthetic electron transport chain.

The oxidized P680^+^, a powerful oxidant (+1.2 V vs SHE),^53^ is then reduced by electron transfer from a redox-active tyrosine residue, which in turn extracts electrons from the oxygen-evolving complex (OEC), a unique Mn_4_CaO_5_ cluster located on the lumenal side of PSII. Sequential oxidation of the OEC drives it through a series of four S-state transitions, culminating in the catalytic oxidation of two water molecules to form molecular oxygen. This process releases four protons into the thylakoid lumen, thereby contributing to the proton motive force that powers the ATP synthesis.

**Figure 3:**
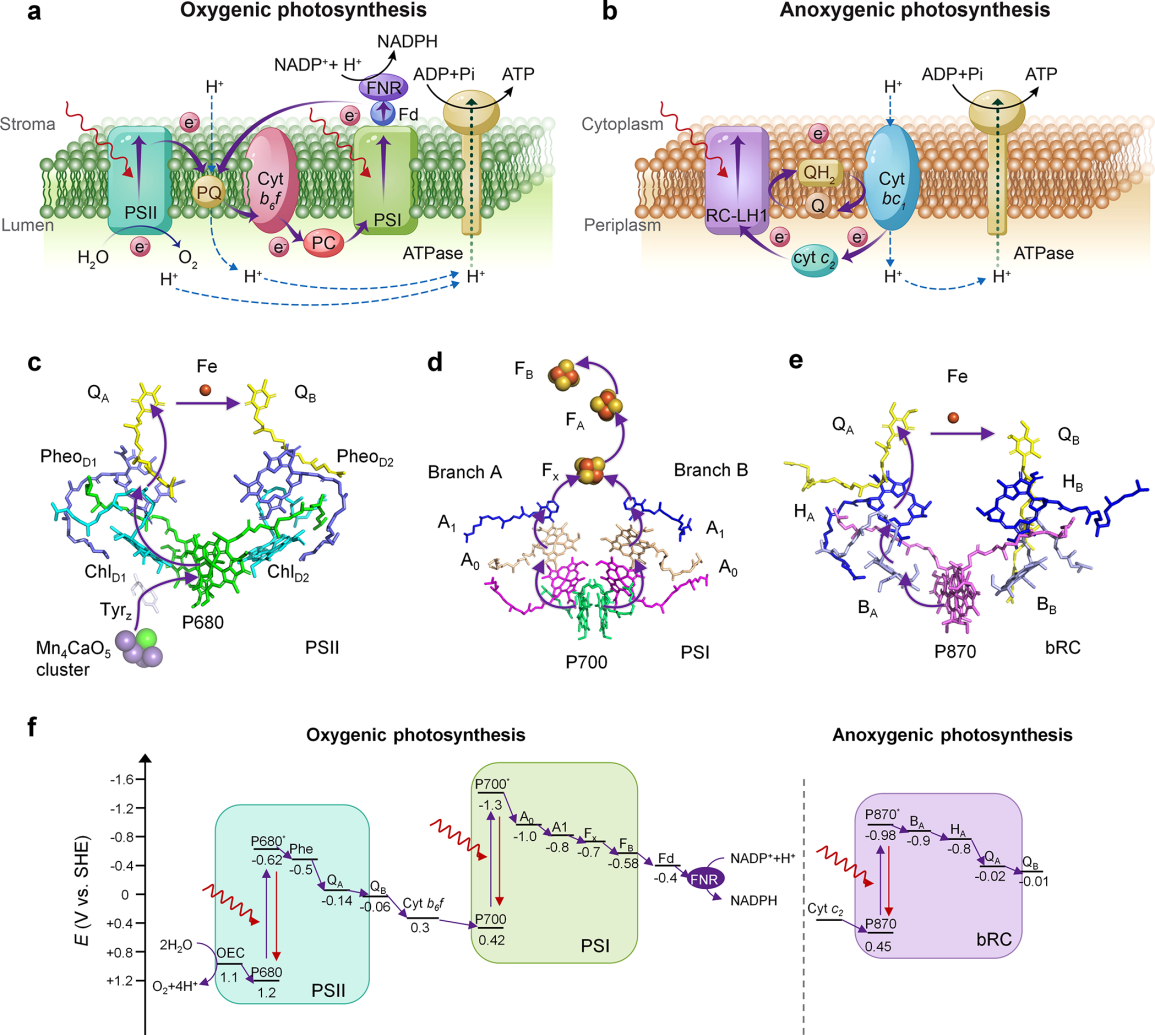
Photosynthesis and photosynthetic proteins. (a) Oxygenic photosynthesis process begins with PSII oxidizing H_2_O to release O_2_, H^+^, and electrons. These electrons travel through an electron transport chain (via plastoquinone (PQ), Cyt *b*_6_ *f*, and plastocyanin (PC)) to PSI. Upon a second light excitation, PSI drives the reduction of NADP^+^ to NADPH via ferredoxin and ferredoxin–NADP^+^ reductase (FNR). Water oxidation and redox cycling of the plastoquinones create a proton gradient across the thylakoid membrane, which drives ATP synthesis. (b) Anoxygenic photosynthesis in purple bacteria starts with charge separation at the reaction center followed by electron transfer to a quinone pool (Q) and to the cytochrome *bc*_1_ complex, where ubiquinol (QH_2_) oxidation is coupled to proton translocation across the membrane, generating a gradient for ATP synthesis. The cycle is completed as reduced cyt *c*_2_ reduce the reaction center. Cofactors arrangement of the electron transfer pathway in (c) PSII, (d) PSI, and (e) bRC. Arrows indicate the electron transfer pathway. PSII PDB 2AXT, PSI PDB 1JB0, bRC PDB 1PCR. (f) Energy level diagram of the cofactors for PSI and PSII, as well as bRC from *Rba. sphaeroides.* Redox potentials are based on the values published in ref 44.

### 2.3 PSI Structure and Function

PSI is a large multisubunit pigment–protein complex that operates downstream of PSII in oxygenic photosynthesis. Its primary function is to convert light energy into chemical energy by driving electron transfer from the high-potential soluble donor plastocyanin to the low-potential iron–sulfur protein ferredoxin that is subsequently used for the production of NADPH (Figure 3a). The core of PSI consists of a heterodimer formed by the PsaA and PsaB subunits, which binds most of the cofactors responsible for light-driven energy conversion.^54,55^ At the center lies the P700 RC, a special pair composed of chlorophyll *a* and chlorophyll *a*′, with a characteristic absorption maximum at 700 nm (Figure 3d). Upon photoexcitation, rapid charge separation occurs at P700, which donates a high-energy electron to the primary acceptor chlorophyll A_0_. The electron is then transferred sequentially via phylloquinone A_1_ to a series of [4Fe–4S] clusters (F_*x*_, F_A_, and F_B_).^56^ This light-induced charge separation generates a potential difference of approximately 1 V between P700 and F_B_ ^57,58^ (Figure 3f).

The highly optimized spatial arrangement and energetic tuning of these cofactors promote ultrafast forward electron transfer while suppressing intraprotein charge recombination. In particular, successive free-energy drops along the electron-transfer chain (Figure 3f) render reverse electron transfer thermodynamically unfavorable, and rapid forward electron-transfer kinetics outcompete charge recombination pathways. Together, these features enable PSI to achieve a quantum yield close to unity.^59^ On the stromal side, electrons are transferred from the F_B_ cluster to soluble ferredoxin, while the oxidized P700^+^ is reduced at the lumenal side by plastocyanin, thereby completing transmembrane electron transfer.

Beyond the PSI RC itself, compartmentalization of the soluble electron carriers also plays a central role in controlling the recombination pathways at the level of the thylakoid membrane. Plastoquinone is confined within the membrane, plastocyanin diffuses within the lumen, and ferredoxin operates on the stromal side. This spatial separation restricts undesired encounters between reduced electron donors and oxidized acceptors, thereby minimizing back-reactions between soluble charge carriers and promoting linear electron flow from PSI to ferredoxin toward the reduction of NADP^+^ to NADPH. Under conditions of excess light or a highly reduced stromal environment, however, electrons can be redirected from reduced ferredoxin back to the plastoquinone pool, activating cyclic electron flow.^59,60^ Although cyclic electron flow does not produce NADPH, it enhances proton translocation across the thylakoid membrane, sustaining ATP synthesis, and contributing to photoprotection.

### 2.4 bRC Structure and Function

The bRC is a transmembrane pigment–protein complex that is central to bacterial anoxygenic photosynthesis. Its primary function is to convert light energy to chemical energy through light-driven charge separation. The bRC is surrounded by light-harvesting complex 1 (LH1), forming the RC-LH1 supercomplex. LH1 consists of a ring-shaped array of pigment–protein subunits that captures incident light and efficiently channels excitation energy to the bRC, thereby substantially enhancing photosynthetic efficiency. Together, these components support a cyclic electron-transfer pathway (Figure 3b) that generates a transmembrane proton gradient used to drive ATP synthesis.

The classic model for bRC structure and function is based on purple bacteria such as *Rhodopseudomonas (Rps.) viridis* and *Rba. sphaeroides*. The bRC core is composed of the L, M, and H subunits, with the L and M subunits forming a heterodimeric scaffold that binds and organizes the redox cofactors into two pseudosymmetrical branches, commonly termed the A- and B-branches^61–63^ (Figure 3e). Despite this symmetry, electron transfer proceeds almost exclusively along the A branch.^64^

The photochemical cycle is initiated by the excitation of the primary electron donor, a specialized bacteriochlorophyll dimer known as the special pair (P870). The excited electron is transferred from P870* to the A-branch bacteriopheophytin (B_A_), then sequentially to the tightly bound quinone Q_A_, and finally to the mobile secondary quinone Q_B_ (Figure 3f). After two photocycles, Q_B_ accepts two electrons and picks up two protons from the cytoplasm to form ubiquinol, which is then diffused into the membrane quinone pool.^65^ The oxidized special pair P870^+^ is subsequently reduced by cytochrome *c*_2_ in the periplasm, resetting the reaction center.

This cyclic, light-driven electron-transfer process establishes a proton motive force across the membrane that powers ATP synthesis, thereby completing the conversion of light energy into biologically usable chemical energy. Overall, photoexcitation and charge separation generate a potential difference of approximately 0.46 V between P870 and Q_B_.^62,66,67^

## 3 Biophotoelectrodes

The fabrication of biophotoelectrodes relies on the isolation of functional photosystems, which requires their extraction from the native membrane environment and poses substantial challenges. Once removed from their integrated biological matrix, photosystems may exhibit markedly reduced stability and may be prone to rapid degradation.^68,69^ In addition, disruption of the membrane’s intrinsically organized charge-transfer pathways and spatial compartmentalization enables charge recombination processes, while eliminating the possibility of conserving energy in the form of a proton gradient. These effects severely limit the operational lifetime and efficiency of biohybrid devices. Consequently, a central objective in the design of biophotoelectrodes is the development of interfaces and matrices that stabilize the photosystems and minimize the charge recombination process while effectively harnessing their native photoconversion efficiency.

Like other redox-active proteins, photosynthetic proteins can exchange electrons with electrodes through either direct electron transfer (DET) or mediated electron transfer (MET).^70^ In DET, electrons are transferred directly between the cofactors of the photosynthetic protein and the electrode. Because efficient tunneling requires a separation distance of less than ∼14 Å, DET is highly sensitive to the orientation of the protein at the electrode interface.^71^ In contrast, MET overcomes this spatial limitation by employing diffusional redox mediators or redox-active polymers that shuttle electrons between the active site and the electrode. As a result, electron transfer becomes largely independent of protein orientation.^72,73^

PSII is of particular interest for the construction of photoanodes (Figure 4a) as it is the only known enzyme capable of catalyzing water oxidation and is therefore essential for supplying electrons in photosystem-based photoelectrochemical devices aimed at solar fuel production. PSI is a highly efficient photoconverter with an exceptional quantum yield. Depending on whether the F_B_ cluster or the P700 reaction center is interfaced with the electrode, PSI can operate in either a photoanodic or photocathodic configuration (Figure 4b). Similarly, bRCs from purple bacteria can function in both photoanode and photocathode architectures depending on their mode of electrical interfacing with the electrode (Figure 4c).

**Figure 4:**
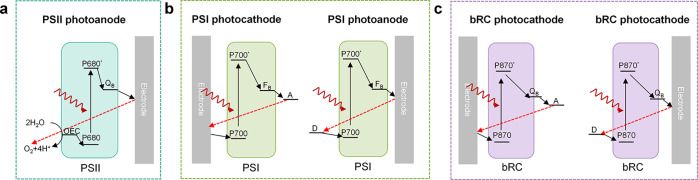
Configurations and electron transfer pathways of biophotoelectrodes. (a) PSII-based photoanode; (b) PSI-based photocathode and PSI-based photoanode; (c) bRC-based photocathode and bRC-based photoanode. Black arrows indicate productive electron transfer pathways contributing to photocurrent generation, whereas red dash arrows represent charge recombination pathways that diminish photocurrent output. A: electron acceptor. D: electron donor.

### 3.1 Configurations and Electron Transfer Pathways

#### 3.1.1 PSII-Based Biophotoelectrode

In a PSII-based photoanode, forward electron transfer is initiated by light-driven water oxidation at PSII, followed by electron transfer to the electrode, resulting in an anodic photocurrent (Figure 4a). Electron transfer from PSII to the electrode can proceed through either DET or MET.^74^ DET configurations maximize energy efficiency because they avoid mediator-associated overpotentials; however, the resulting photocurrents are generally lower than those achieved under MET conditions. Among mediators, the use of 2,6-dichloro-1,4-benzoquinone, a high-potential freely diffusing quinone, typically yields the highest photocurrents, albeit at the expense of a substantial overpotential.^74,75^ Beyond quinones, *in vivo* studies suggest that phenazines may function as low-potential electron shuttles for PSII, opening opportunities to improve the energy efficiency of photocurrent generation.^76^

In biophotoelectrodes, redox-active mediators are often immobilized on polymer. Representative examples include polymers functionalized with benzoquinones,^77,78^ phenothiazines^79^ and Os-complexes,^80–82^ which simultaneously facilitate electron transfer and provide a matrix for PSII immobilization. Alternative coimmobilization strategies have also been explored, such as PSII–ferricyanide intercalated architectures.^83^

Under both DET and MET conditions, electron extraction from PSII most commonly occurs via the Q_B_ site, as evidenced by the strong photocurrent inhibition upon addition of herbicides that specifically bind this site.^82^ Nevertheless, electron transfer from PSII can also bypass Q_B_, proceeding directly from the Q_A_ site^69,84^ or from peripheral chlorophyll pigments, potentially enabling greater thermodynamic gains.^85^ Irrespective of the electron extraction pathway, PSII-based biophotoanodes evolve O_2_ as a product of water oxidation. Because the oxygen reduction reaction proceeds relatively slowly, the O_2_ generated at the photoanode contributes only minimally to charge recombination under the high anodic potentials typically applied in MET-based systems.^86^ In contrast, under DET conditions or when low-potential mediators are employed, photoinduced oxygen reduction by antenna chlorophyll pigments can compete with productive charge transfer at the enzyme–electrode interface, thereby decreasing photocurrent output.^87,88^

#### 3.1.2 PSI-Based Photoelectrode

Although PSI lacks intrinsic catalytic activity, it is highly attractive for biophotoelectrochemical applications because of its exceptionally rapid intraprotein electron-transfer kinetics^29,30,89^ and its large photoinduced charge separation. PSI is most commonly employed in photocathode configurations, in which the electrode supplies electrons, typically through redox mediators, to the donor side of PSI (P700/P700^+^) (Figure 4b). Upon photoexcitation, charge separation drives electron transfer through the PSI cofactor chain to the terminal [4Fe-4S] clusters, F_A_ and F_B_, from which electrons are transferred to a soluble electron acceptor or charge carrier.

Common mediators used to shuttle electrons from the electrode to P700 include cytochrome *c* and Os-complex-modified polymers, while methyl viologen (MV^2+^) is frequently employed to extract electrons from the terminal F_B_ cluster. The redox potential of these artificial electron carriers are well matched to those of the natural PSI electron carriers plastocyanin and ferredoxin, respectively. However, when MV^2+^ is used as the sole electron acceptor, sustained photocathodic currents are often not achieved because the reduced radical cation (MV^+•^) is readily reoxidized at the electrode surface, resulting in charge recombination. To suppress this back-electron transfer pathway, molecular oxygen can be introduced as a sacrificial electron acceptor that rapidly oxidizes MV^+•^, thereby trapping the photogenerated electron in the products of the oxygen reduction reaction (Figure 5b). Although this strategy effectively enforces unidirectional electron flow and enables quantitative evaluation of electron-transfer kinetics in PSI-based biophotoelectrodes, it is unsuitable for practical solar-energy conversion because the energy is dissipated as heat and the electrons are ultimately returned to water through oxygen reduction (Figure 5c).

**Figure 5:**
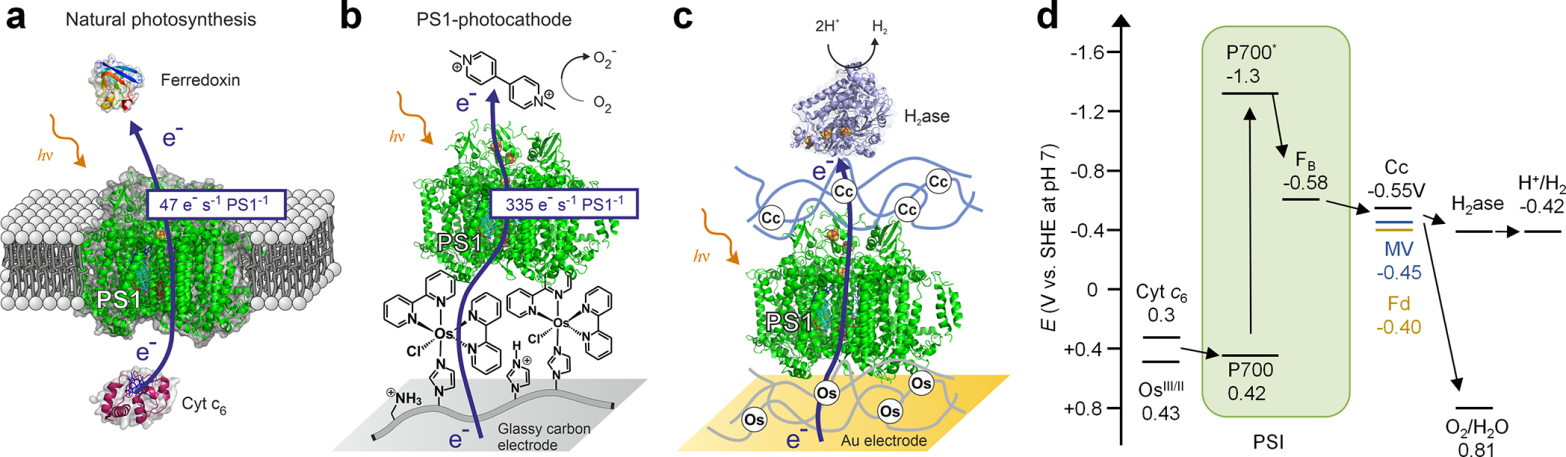
Electron-transfer pathways in natural photosynthesis compared to PSI-based photocathode for photocurrent and hydrogen generation. (a) In natural photosynthesis, directional electron flow is achieved through spatial separation of electrons and holes into distinct membrane compartments, thereby minimizing charge recombination. (b) In PSI-based photocathode, the overall electron transfer rate through PSI can exceed that observed *in vivo* through efficient electronic coupling mediated by artificial redox polymers, while molecular oxygen acts as a sacrificial electron acceptor to suppress back-electron transfer. For clarity, the osmium-complex-modified polymer and PSI are illustrated as a single layer; in practice, the polymer–PSI assembly forms micrometer-thick films consisting of randomly oriented, yet homogeneously distributed PSI complexes embedded within the redox-polymer matrix. Reproduced with the permission from ref 89. Copyright 2014 Wiley. (c) Light-driven hydrogen evolution achieved by coupling PSI with *D. gigas* hydrogenase (H_2_ase) in layered configuration. Electron transfer between the two proteins is mediated by two redox polymers: an osmium-complex-modified polymer with a relatively positive redox potential and a cobaltocene-functionalized polymer with a more negative potential.^90^ Adapted with the permission from ref 90. Copyright 2017 Wiley. (d) Combined energy diagram for the three PSI-electron transfer pathways shown in panel (a–c).

Productive light-to-energy conversion in a PSI-based photocathode can be achieved through spatial compartmentalization of electron mediators, thereby mimicking the membrane-separated architecture of natural photosynthesis (Figure 5a). Gorton et al. demonstrated this principle using a layer-by-layer electrode assembly in which a low-potential Os-complex-modified polymer was first deposited on the electrode, followed by a PSI layer, and finally a cobaltocene-modified polymer incorporating a hydrogenase (Figure 5c).^90^ This architecture prevents the reduced low-potential electron carrier (cobaltocene) from directly interacting with the electrode, the Os complex, or the donor side of PSI, thereby suppressing charge recombination pathways. As a result, directional electron transfer from PSI to the hydrogenase is enabled, leading to light-driven hydrogen evolution. Although the resulting photocurrents were modest, this pioneering demonstration highlighted the central importance of compartmentalization and rational electrode design in minimizing charge recombination and enabling productive solar-to-hydrogen conversion.

PSI-based photoelectrodes can also be configured as photoanodes. In this configuration, photoexcitation drives charge separation within PSI, after which electrons are extracted from the terminal F_B_ site to the electrode, while an external electron donor regenerates P700^+^ (Figure 4b). Anodic photocurrents have been demonstrated using dichloroindophenol (DCPIP) as an electron mediator in combination with ascorbate (Asc) as a sacrificial electron donor.^91,92^ In this system, ascorbate suppresses charge recombination by reducing the oxidized mediator, thus preventing its reduction at the electrode surface. An alternative PSI-based photoanode architecture employs PSI immobilized as a monolayer on a PQQ-modified electrode, coupled to glucose oxidase embedded within an osmium complex-modified redox polymer that serves as a unidirectional electron donor to PSI. In this case, charge recombination is minimized through the irreversible oxidation of glucose, which functions as a sacrificial electron donor and enables sustained anodic photocurrents.^93^ The onset potential for photocurrent generation can be shifted to more negative values and thus allow greater recovery of the photogenerated energy at PSI, by replacing the PQQ mediator with lower-potential mediators such as viologen-modified polymers that shuttle electrons from the terminal F_B_ site to the electrode.^94^

More commonly, PSI-based photoanodes are implemented in dye-sensitized solar cell (DSSC) type architectures, in which a semiconductor electrode directly accepts electrons from the F_B_ site.^95,96^ Such configurations can substantially reduce charge recombination losses and improve photocurrent generation without requiring sacrificial electron donors.

#### 3.1.3 bRC-Based Photoelectrode

Like PSI, the purple bacterial reaction center can be configured as either photoanodes or a photocathodes, although they are more commonly employed as photocathodes (Figure 4c). In the photocathode configuration, photoexcitation of the special pair (P870) initiates electron transfer through the bRC, ultimately reducing a quinone at the Q_B_ site. The resulting photo-oxidized P870^+^ is subsequently reduced by electrons supplied from the electrode, either via DET or MET. Common redox mediators used for MET include cyt *c* ^97^ or Os-complex-modified polymers.^98^ On the acceptor side, photoinduced electron transfer reduces an exogenous quinone at the Q_B_ site to quinol, which subsequently dissociates from the binding pocket into the electrolyte, carrying electrons and protons. The exogenous quinone electron acceptor is typically a soluble benzoquinone such as Q_0_ added to the solution.^97,99,100^ Nevertheless, in some bRC preparations, native water-insoluble quinones such as Q_10_ ^101^ (for example in RC–LH1 complexes) are retained during protein purification and may serve as endogenous electron acceptors, subsequently transferring electrons to the exogenous soluble Q_0_.

The reduced quinol species (e.g., Q_0_H_2_) can be reoxidized at the electrode surface, resulting in charge recombination. However, because the Q_0_/Q_0_H_2_ redox couple undergoes coupled two-electron/two-proton transfer and the potential difference between the donor and acceptor sides of bRC is moderate, quinol oxidation, and thus charge recombination, proceed relatively slowly. Consequently, unlike PSI-based photocathodes, which often require sacrificial electron acceptors to suppress rapid recombination, bRC-based photocathodes can sustain photocurrents without sacrificial acceptors.^100^ The extent of charge recombination can be assessed by potential step voltammetry, where it manifests as an anodic dark current upon cessation of illumination.^99^ Semiconductor and metal oxide electrodes, such as indium tin oxide (ITO), are frequently employed to further suppress charge recombination.

When the bRC assembly is configured to enable electron transfer from its acceptor side (Q-side) to the electrode via either DET or MET, a bRC-based photoanode can likewise be constructed^102–106^ (Figure 4c). In such configurations, photoanodic currents have been demonstrated using reduced cyt *c* as the electron donor.^102,103^

Additional advantages of bRC-based photoelectrodes include their ability to harvest near-infrared light for photocurrent generation^107^ and their oxygen-independent operation, as neither O_2_ evolution nor O_2_ consumption is involved in the reaction cycle. The absence of oxygen-related side reactions contributes to the excellent operational stability of the bRC-based systems. Although bRCs do not possess specialized functionalities such as the water-oxidation catalysis of PSII or the exceptionally high photovoltage of PSI, their robustness, moderate reductive force, and catalytic quinol production facilitate efficient and sustained photocurrent generation, making them highly attractive and widely used components in biophotoelectrochemical devices.^108^

### 3.2 Performance Metrics of Photoelectrochemical Systems

Benchmarking and improving photosystem-based photoelectrochemical systems for solar fuel and electricity generation requires characterization of their performances, which is commonly evaluated using metrics such as photocurrent density, overpotential, quantum efficiency, energy efficiency, and operational stability, which collectively determine both their potential for practical application.

#### 3.2.1 Photocurrent

Most studies on biophotoelectrodes report photocurrent density (*J*_photo_ in A cm^–2^) as the primary performance metric (Figure 6), as it directly reflects the activity of the photosynthetic proteins and the efficiency of light-driven electron transfer. Photocurrents depend on numerous parameters, including the applied electrode potential, photosystem loading, intrinsic photosystem activity, electron-transfer rates of soluble or immobilized redox mediators, heterogeneous electron-transfer kinetics at the electrode interface, light intensity and spectral profile, concentrations of electron donors and acceptors, and the extent of charge recombination.^74,86^

**Figure 6:**
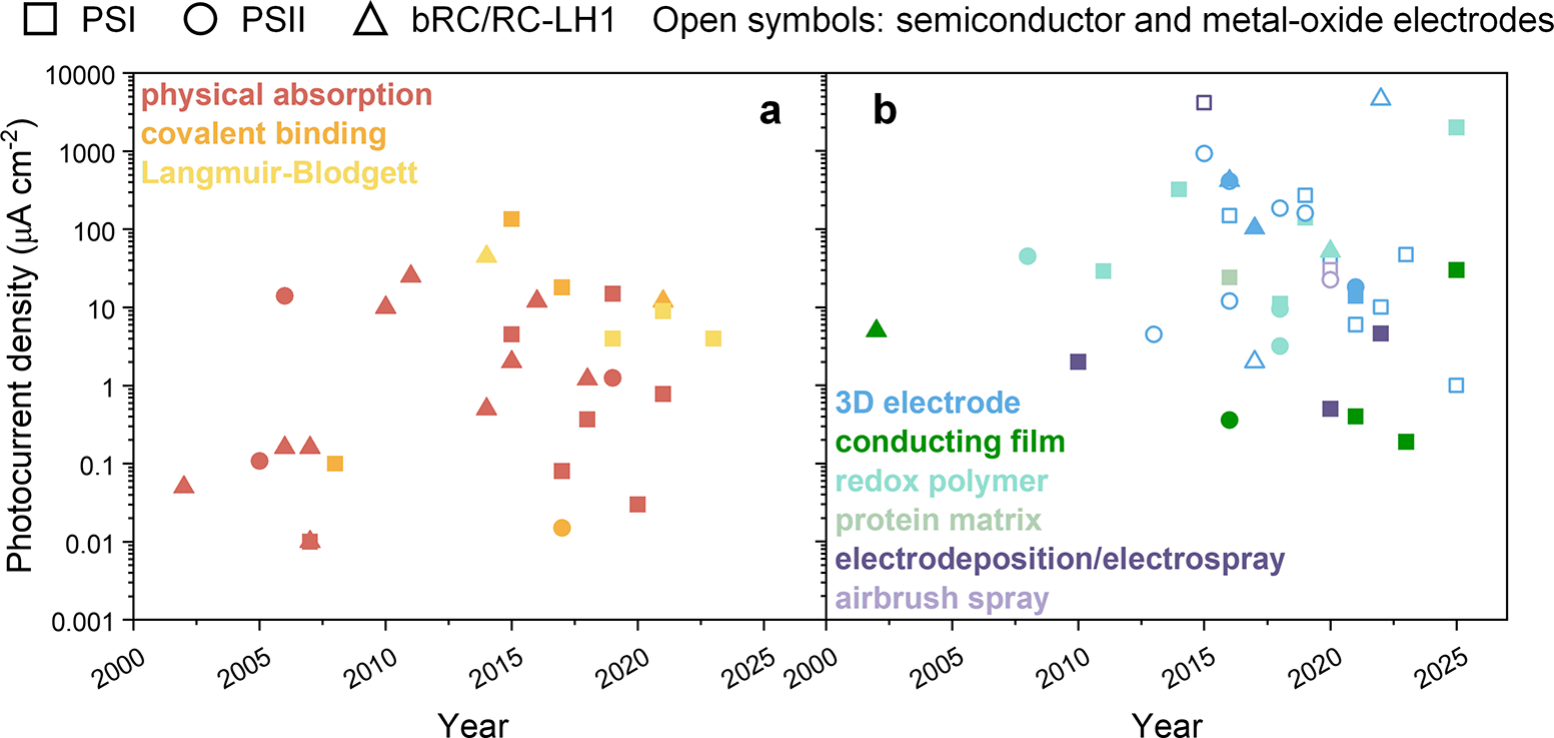
Progress in photocurrent densities achieved with photosystem-based biophotoelectrodes fabricated using different immobilization strategies. (a) Photocurrent densities obtained from photosystem-based electrodes prepared by physical adsorption, covalent attachment, or Langmuir–Blodgett deposition, which typically produce monolayer architectures. (b) Photocurrent densities obtained from photosystem-based electrodes fabricated using three-dimensional electrode architectures, conductive films, redox-active polymers, protein matrices, electrodeposition, electrospray deposition, and airbrush spray techniques, which generally produce multilayer or multilayer-equivalent assemblies. PSI-based electrodes are indicated by squares,^89,109–143^ PSII-based electrodes by circles,^78,80,81,144–155^ bRC-based electrodes by triangles.^97–100,104,156–170^ Semiconductor-based and metal-oxide-based electrodes are represented by open symbols. The plotted photocurrent densities correspond to the total measured photocurrent, including contributions from the electrode materials themselves, which can be substantial for semiconductor and metal-oxide-based systems. Details and references for each data point are provided in Table S1 in the Supporting Information.

The evolution of photocurrent performance over time highlights the advances in electrode design and immobilization strategies. Early pioneering studies, in which photosynthetic proteins were immobilized directly on electrode surfaces typically resulting in protein monolayers, produced photocurrent densities ranging from approximately 10 nA cm^–2^ to 100 μA cm^–2^ (Figure 6a). In these systems, performance was primarily limited by the loading of photosynthetic proteins, largely independent of the specific photosystem employed. Over the past decade, increasing emphasis has been placed on strategies that enable substantially higher protein loadings, resulting in photocurrent densities commonly spanning from 1 μA cm^–2^ to 10 mA cm^–2^, again largely independent of the photosystem used (Figure 6b). In parallel, the adoption of semiconductor and metal-oxide electrodes capable of suppressing charge recombination has further contributed to the steady increase in photocurrent performance.

An important prerequisite for a meaningful comparison between different systems is the use of a standardized methodology for photocurrent determination. Regardless of whether photoanode or photocathode configurations are employed, photocurrent is typically monitored as a function of time under an applied constant potential while alternating between dark and illuminated conditions^171^ (Figure 7a). The measured current was normalized to the geometric electrode area (illuminated surface area) to obtain the photocurrent density. Under illumination, the recorded current consists of both the light-induced photocurrent and a background current originating from capacitive charging and Faradaic reactions of redox-active species in the electrolyte that occur independently of illumination. The true photocurrent is therefore determined by subtracting the dark current from the total current measured under illumination.

Accurate identification of the origin of the photoresponse further requires systematic control experiments. Blank electrodes are used to evaluate the intrinsic photoactivity of the electrode materials themselves. Controls performed under illumination comparing active biophotoelectrodes and protein-free electrodes clarify the specific contribution of the photosynthetic proteins to photocurrent generation.^172^ The role of the photosystems can be further verified using inactive protein preparations or selective inhibitors.^69^ Control experiments involving electron donors and acceptors are also essential to confirm productive electron transfer between the photosynthetic proteins and the electrochemical system. Together, these experiments ensure that the measured photocurrent originates from the intended biohybrid photoelectrochemical processes rather than from experimental artifacts or parasitic reactions, such as current changes due to temperature increase upon illumination.

Upon light exposure, the photocurrent response often features a peak current (*j*_peak_) followed by a decay that either stabilizes at a steady-state value (stable current, *j*_stable_) or continues to decrease over time.This decay can result from several factors, including charge recombination, buildup of a diffusion layer in the case of mass transport limitations of the terminal electron donor or acceptor, desorption of photosynthetic proteins from the electrode surface, and irreversible protein degradation.

As illustrative examples, PSII-modified electrodes typically function as photoanodes and generate positive photocurrents.^69^ When PSII is immobilized as a monolayer and operated via direct electron transfer, the resulting photocurrent is often small, leading to a pronounced contribution from the background current, as evident in dark–light cycling experiments (Figure 7b). In contrast, RC-LH1-modified electrodes represent a classical photocathode system yielding negative photocurrents.^170^ Under stationary conditions, photocurrent typically decays during illumination due to depletion of the oxidized quinone used as soluble electron acceptor, while charge recombination is manifested as a reverse (positive) current upon switching off the light due to reoxidation of the quinol product at the electrode surface (Figure 7c). Both recombination and mass-transport limitations can be mitigated by forced convection, enabling the establishment of a steady-state photocurrent provided that the electrode architecture remains stable. If irreversible degradation processes occur, however, the photocurrent will continue to decline over time.

**Figure 7:**
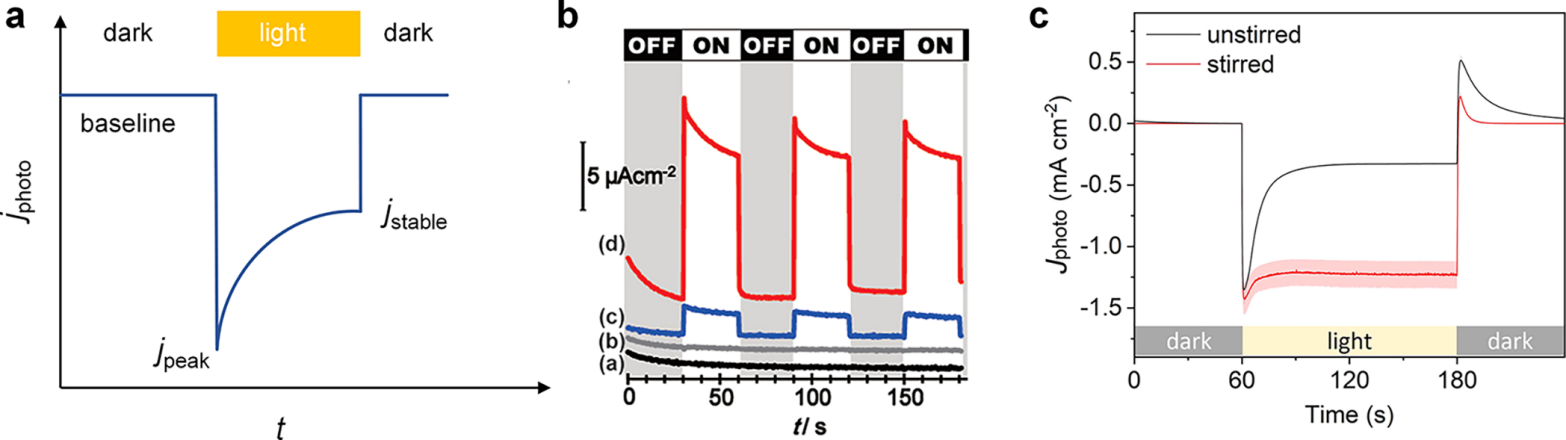
Photocurrent generation in biophotoelectrochemical systems. (a) Schematic of a typical cathodic photocurrent recorded under alternating light–dark cycles at constant applied potential. (b) Anodic photocurrent generated by a PSII-modified mesoporous ITO electrode. Reproduced with the permission from ref 69. Copyright 2012 American Chemical Society. (c) Cathodic photocurrent generated by an RC-LH1/cyt *c* modified IO-mITO electrode using Q_0_ as soluble electron acceptor, recorded in the presence and absence of stirring. Reproduced with the permission from ref 170. Copyright 2012 Wiley.

#### 3.2.2 Onset Potential

The onset potential (in V vs the standard hydrogen electrode) for photocurrent generation is a key performance parameter because it defines the maximum achievable energy conversion efficiency of a device. This reflects the energetic losses associated with electron transfer between the photosynthetic proteins and the electrode. In biophotoelectrochemistry, the onset potential is commonly defined as the potential at which the photocurrent rises measurably above the background (dark) current.

Experimentally, the onset potential is typically determined by potential step voltammetry in which the photocurrent is plotted as the function of applied potential^80^ (Figure 8a), by chopped light voltammetry^123^ (Figure 8b), or by comparing cyclic voltammograms (CVs) recorded in the dark and under illumination at slow scan rates^89,99,145^ (Figure 8c). When the photocurrent increases sharply with applied potential, the onset potential can be identified unambiguously, as illustrated in Figure 8a. In contrast, systems exhibiting a gradual photocurrent increase yield onset potential values that are inherently more subjective and less well-defined (Figure 8b). In cases where a well-defined photocatalytic wave is observed, extracting its position from the first derivative of the current–potential curve (Figure 8c) is preferable to using an onset potential, as this approach provides a more robust and reproducible metric.

#### 3.2.3 Quantum Efficiency

Although isolated photosynthetic proteins exhibit near-unity quantum efficiencies for primary charge separation, biophotoelectrodes typically display substantially lower overall quantum efficiencies because of optical losses, incomplete protein wiring, charge recombination, and kinetic limitations at the electrode interface. Two quantum efficiency metrics are commonly used to evaluate biophotoelectrodes:

##### 3.2.3.1 The External Quantum Efficiency (EQE)

also referred to as the incident photon-to-current efficiency (IPCE), quantifies the fraction of incident photons converted into photocurrent.^172^ The external quantum efficiency is defined as

$$\mathrm{EQE}=\frac{j_{\mathrm{photo}}}{e\times \Phi_{\mathrm{inc}}}$$

where *j*_photo_ is the photocurrent density (A cm^–2^), *e* is the elementary charge (1.602 × 10^–19^ C), Φ_inc_ is the incident photon flux density (photons cm^–2^ s^–1^). The incident photon flux is calculated from the spectral irradiance and wavelength distribution of the illumination source. EQE therefore reflects the overall efficiency of the device in converting incident light into electrical current, including optical losses arising from reflection and incomplete absorption.

##### 3.2.3.2 The Internal Quantum Efficiency

(IQE) quantifies the fraction of absorbed photons converted into photocurrent.^91^ The internal quantum efficiency is defined as

$$\mathrm{IQE}=\frac{j_{\mathrm{photo}}}{e\times \Phi_{\mathrm{abs}}}$$

where the Φ_abs_ is the absorbed photon flux density (photons cm^–2^ s^–1^). Unlike EQE, IQE excludes photons that are not absorbed by the biophotoelectrode and therefore reflects the efficiency with which absorbed photons are converted into charge carriers.

The absorbed photon flux can be estimated from the spectral photon flux and the wavelength-dependent optical transmission of the biophotoelectrode according to

$$\Phi_{\mathrm{abs}}=\int \Phi_{\mathrm{inc}}\left(\lambda \right)\left(1-T\left(\lambda \right)\right)\;d\lambda$$

where Φ_inc_(λ) is the wavelength-dependent incident photon flux density (photons cm^–2^ s^–1^), *T*(λ) is the wavelength-dependent transmission of the biophotoelectrode, λ is wavelength (nm).

Because IQE is normalized to the number of absorbed photons, it provides a more direct measure of the efficiency of photochemical charge generation and electron extraction within the biohybrid electrode architecture.

**Figure 8:**
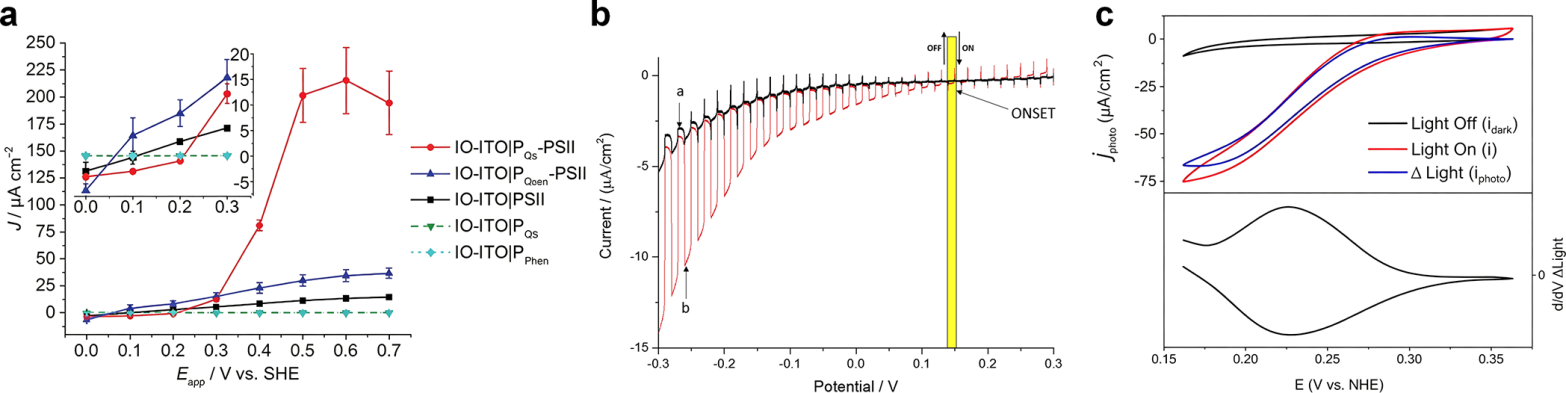
Onset potential determination for photocurrent generation. (a) Photocurrent as a function of the applied potential for PSII photoanodes determined by stepped potential chronoamperometry. Reproduced with the permission from ref 80. Copyright 2016 The Royal Society of Chemistry. (b) Chopped light voltammetry of PSI photocathode. Reproduced with the permission from ref 123. Copyright 2016 Elsevier. (c) Cyclic voltammetry of the RC-LH1 photocathode in the dark and in the light (top) and derivative of the catalytic wave (bottom). Reproduced with the permission from ref 99. Copyright 2016 Wiley.

#### 3.2.4 Energy Efficiency for Electricity and Solar Fuels Production

##### 3.2.4.1 Electricity Generation

Biophotovoltaic devices integrate photosynthetic proteins into electrode architectures to convert solar energy into electricity. The overall light-to-electricity conversion efficiency (η) of the biophotovoltaic device is determined from the short-circuit photocurrent density (*J*_sc_, in A cm^–2^), the open-circuit photovoltage (*V*_oc_, in V), the fill factor of the cell (ff), and the incident light intensity (*P*_in_, in W cm^–2^):^173^

$$\eta =\frac{J_{\mathrm{sc}}V_{\mathrm{oc}}ff}{P_{\mathrm{in}}}$$

The fill factor is defined as the ratio between the maximum obtainable power density (*P*_max_, W cm^–2^) and the product of *V*_oc_ and *J*_sc_:

$$ff=\frac{P_{\max}}{J_{\mathrm{sc}}V_{\mathrm{oc}}}$$

The fill factor provides a measure of energy losses within the device, including those arising from charge recombination, kinetic limitations, and resistive processes, and therefore serves as an important indicator of overall device performance. In this respect, the fill factor in biophotovoltaic systems is conceptually analogous to the ability of natural photosynthetic systems to suppress recombination and other dissipative pathways in order to sustain productive directional electron transfer. Consequently, achieving high energy-conversion efficiencies (η) requires biophotoelectrodes capable of generating large photocurrent densities while simultaneously minimizing overpotentials, charge recombination, and other energy-loss processes.

##### 3.2.4.2 Solar Fuels Production

The performance of solar-to-fuel conversion systems is commonly evaluated using the solar-to-fuel conversion efficiency (η_STF_), which represents the fraction of incident solar energy stored as chemical energy in the fuel product. It can be expressed as^174^

$$\eta_{\mathrm{STF}}=\frac{\mathrm{chemical energy output}}{\mathrm{solar energy input}}=\frac{\Delta G\times r}{P_{\mathrm{in}}\times S}$$

where Δ*G* is the Gibbs free energy of the reaction associated with fuel formation (J mol^–1^), *r* is the fuel production rate (mol s^–1^), *P*_in_ is the incident light intensity (W cm^–2^), and *S* is the illuminated area (cm^2^). This definition applies to full-cell measurements of endergonic fuel forming reactions, typically coupling water oxidation to fuel production, in the absence of an externally applied bias.

For biophotoelectrochemical systems, the solar-to-fuel efficiency is related to the photocurrent density according to^174–177^

$$\eta_{\mathrm{STF}}=\frac{J_{\mathrm{photo}}\times \Delta E\times \eta_{F}}{P_{\mathrm{in}}}$$

where *J*_photo_ is the photocurrent density (A cm^–2^), Δ*E* is the thermodynamic potential difference associated with the fuel-forming redox reaction (V), and η_F_ is the faradaic efficiency.

The Faradaic efficiency describes the selectivity of the process by quantifying the fraction of transferred electrons that contribute to formation of the desired fuel product rather than to competing side reactions:

$$\eta_{F}=\frac{\mathrm{experimental fuel production}}{\mathrm{theoretical fuel production derived from}\;J_{\mathrm{photo}}}$$

Thus, achieving high solar-to-fuel conversion efficiencies requires biophotoelectrodes that simultaneously deliver high photocurrent densities, low energetic losses, and high Faradaic efficiencies toward the target fuel products.

For biophotoelectrochemical systems that require an external bias (*V*_bias_ in V) to drive thermodynamically uphill fuel-forming reactions, the applied-bias photon-to-current efficiency (ABPE) should be used to evaluate overall device performance under operational conditions.

$$\mathrm{ABPE}=\frac{J_{\mathrm{photo}}\left(\mathrm{\Delta E}-V_{\mathrm{bias}}\right)}{P_{\mathrm{in}}}$$

#### 3.2.5 Stability

Stability remains a primary challenge for the practical application of biophotoelectrodes.^178^ It is often expressed as its half-life, which is defined as the time required for the photocurrent to decay to 50% of its initial value under continuous illumination. Reported half-lives vary significantly across different systems, PSII-based photoelectrodes typically exhibit half-lives ranging from minutes to tens of minutes,^78,80,146,151,154^ PSI-based systems from minutes to several hours,^89,139^ and bRC-based electrodes on the order of hours.^99,170^

In addition to half-life, stability is also evaluated through other metrics. These include the percentage of photocurrent retained after a fixed period under continuous illumination^119,123^ or intermittent light–dark cycles,^122,124,126,136^ the total functional duration of the electrode,^155,162,168^ or the storage performance.^111,170^ Another informative measure is the turnover number (TON), defined as the total number of catalytic cycles per photosystem.^155^ This value can be expressed as the total charge derived from the integrated photocurrent over the electrode’s operational lifetime until activity is fully lost.^99^ Determination of TON requires quantification of the amount of photosystem immobilized on the electrode, which can be estimated from optical absorbance measurements when the electrode is sufficiently transparent, and the photosystem loading is high enough to produce a detectable signal.^99^

Reliable determination and comparison of performance metrics, including photocurrent density, onset potential, quantum efficiency, energy conversion efficiency, and operational stability, require standardized measurement protocols, including well-defined illumination conditions and calibrated light spectra comparable to those used in conventional photovoltaic characterization. However, such broadly accepted standards have not yet been established for photosystem-based biophotoelectrodes.

### 3.3 Protein Immobilization Strategies

Over the past two decades, the field has developed and refined a wide range of immobilization strategies (Figure 9) aimed at constructing high-performance photosystem-based electrodes. Early efforts primarily focused on approaches such as physical adsorption, covalent attachment, and Langmuir–Blodgett assembly on flat electrodes leading to monolayers of the immobibilized photosystems. These methods were particularly valuable for proof-of-concept studies, enabling control over protein orientation, preservation of structural integrity, and optimization of interfacial electron transfer. However, immobilization on planar electrodes is inherently limited by low protein loading, which restricts achievable photocurrent densities (Figure 6a). In recent years, strategies have shifted toward the use of three-dimensional (3D) electrode architectures and the entrapment of photosynthetic proteins within micrometer-thick, redox-active films. These advances substantially increase protein loading and have led to markedly enhanced photocurrent densities, in some cases reaching thresholds in the mA cm^–2^ range that are relevant for practical energy-conversion applications (Figure 6b).

**Figure 9:**
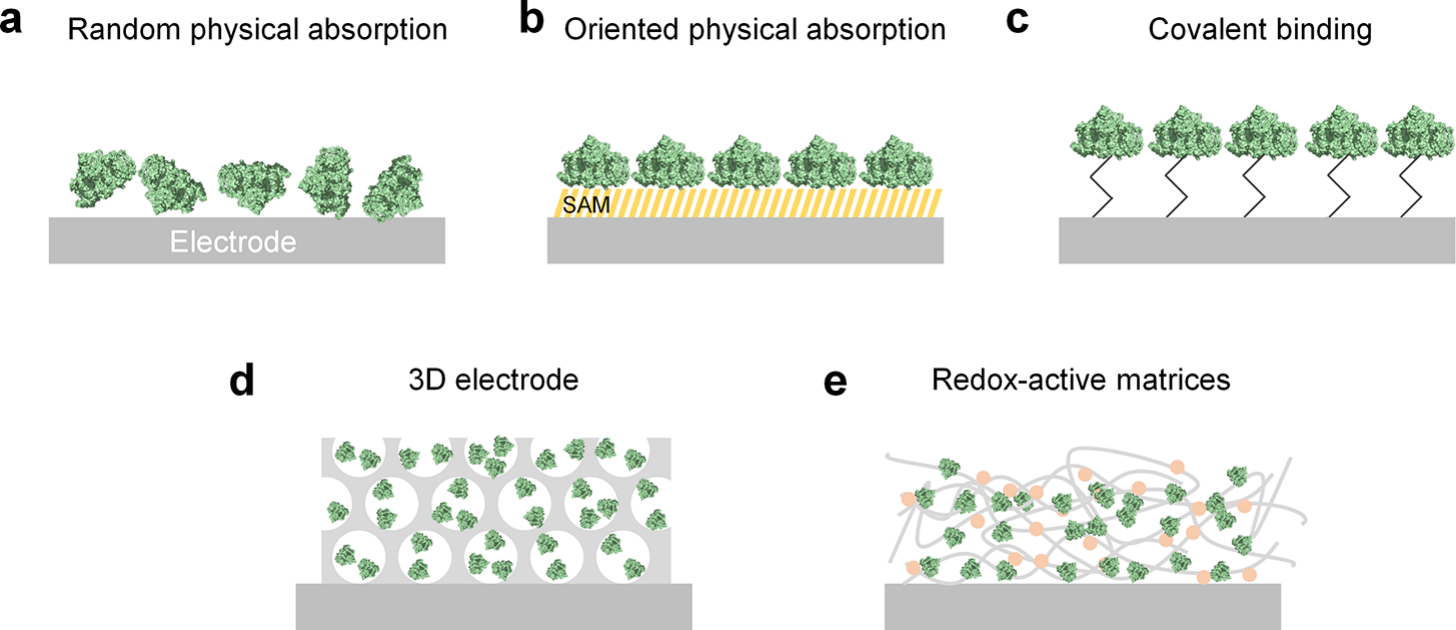
Strategies for immobilizing photosynthetic proteins on electrode surfaces. (a) Random adsorption onto unmodified electrodes. (b) Preferentially oriented adsorption on self-assembled monolayer (SAM)-modified electrodes. (c) Covalent immobilization through chemical surface functionalization. (d) Integration within three-dimensional porous electrode architectures to increase protein loading. (e) Encapsulation within redox-active matrices that simultaneously provide immobilization and electrical wiring of the photosynthetic proteins.

A broad range of electrode materials, including noble metals such as Au, metal oxides, and diverse carbon-based substrates, have been engineered through nanostructuring, surface functionalization, and the incorporation of molecular monolayers. This section reviews the fundamental immobilization strategies employed in biophotoelectrochemistry, highlighting how the synergy between immobilization methods, protein selection or engineering, and electrode material design is crucial for optimizing the photosystem–electrode interface. For a detailed discussion of specific immobilization techniques, readers are referred to recent comprehensive reviews on the topic.^73,179–181^

#### 3.3.1 Physical Adsorption

Early studies used unmodified metallic electrodes for the immobilization of RC-LH1 complexes (Figure 9a). RC-LH1 from bacterium *Rba. sphaeroides* was directly absorbed on a bare Au electrode,^166^ and similar approaches were reported for RC-LH1 from *Rhodoblastus acidophila.* ^162^ In these systems, photocurrents were only observed in the presence of soluble redox mediators, indicating negligible DET between the proteins and unmodified electrodes. This limitation was attributed to the lack of controlled protein orientation and the excessive distance between the electrode surface and redox-active cofactors within the proteins.

Favorable orientation of photosynthetic proteins on electrodes can be promoted through electrostatic interactions with self-assembled monolayers (SAMs) on gold electrodes^182,183^ (Figure 9b). For example, Ciornii et al. used ruthenium hexaminethiol-modified gold surfaces to direct the assembly of PSI, ensuring that its luminal side faced the electrode through electrostatic attraction to the positively charged ruthenium complexes.^123^ Electrical communication between PSI and the electrode can be improved by incorporating carboxyl-modified C70-fullerenes.^123^ PSI self-assembled on SAMs of [6,6]-phenyl-C61-butyric acid on gold, inducing a preferred orientation compatible with efficient interfacial electron transfer.^184^ In a different approach, Gordiichuk et al. used phage display to select small peptides that selectively bind the stromal side of PSI, thereby orienting the terminal F_B_ cluster toward the electrode modified with dihydroxyacetone phosphate.^185^

Controlled orientation of the photosynthetic protein on electrode surfaces has also been achieved through genetic engineering, combined with affinity-based immobilization. Bacterial reaction centers from *Rba. sphaeroides* ^186^ and PSII from *Synechococcus elongatus* ^144,145^ engineered with polyhistidine tags were immobilized as oriented monolayers on Ni-nitrilotriacetic acid (Ni-NTA)-modified gold electrodes.

Graphene-based materials have also been used for photosystem immobilization through physical adsorption. PSI from *T. elongatus* was absorbed onto π-system-modified graphene interfaces via π–π-stacking interactions between polycyclic aromatic compounds and graphene.^114,115^ Single layer and multilayer graphene electrodes were also used for the immobilization of bRCs from *Rba. sphaeroides*.^168^ Uniform orientation of PSI on single-layer graphene was achieved using cytochrome *c*_553_, which is His_6_-tagged and bound to a graphene surface modified with a pyrene-based SAM terminated with Ni-NTA groups. This assembly directed the donor side of PSI toward the electrode, enabling efficient electron transfer.^121^ Similarly, Izzo et al. genetically engineered PSI with an His_6_-tag at the N-terminus of the stromal PsaD subunit and interfaced it with a single layer graphene using pyrene-nitrilotriacetic acid SAM.^131^

Beyond graphene, carbon nanotubes have been applied to photosystem immobilization. PSI was genetically modified to include a peptide sequence within the PsaE subunit that exhibits high affinity for carbon nanotubes, enabling conjugation while orienting the electron acceptor side toward the single-walled carbon nanotube (SWCNT).^120^ In another study, PSI and PSII extracted from spinach were self-assembled on nitrogen-doped carbon nanotubes via an electrostatic interaction. Below pH 5, the SWCNTs were positively charged and promoted spontaneous immobilization of the negatively charged stroma side of photosystems, resulting in a favorable orientation for electron transfer without the need for additional electrode modification.^153^

In addition, the amphiphilic nature of membrane proteins has been exploited to preassemble densely packed PSI monolayer with defined anisotropic orientation at a liquid–gas interface followed by their transfer on electrode surfaces by Langmuir–Blodgett deposition.^125,134,140^

#### 3.3.2 Covalent Binding

Controlled orientation of photosynthetic proteins on electrode surfaces can be achieved through covalent immobilization strategies (Figure 9c). For example, PSI was covalently attached to a gold electrode in a defined orientation to promote efficient electron transfer.^110^ In this approach, the gold surface was first modified with an amine-terminated self-assembled monolayer, which was subsequently reacted with terephthaldialdehyde. The aldehyde-functionalized SAM then formed covalent imine linkages with lysine residues on PSI, resulting in preferential orientation of the complex with the P700 RC positioned proximal to the electrode surface and thereby facilitating electron transfer.

Similar approaches have been implemented using π-conjugated carbon materials. Graphene modified with pyrene butyric acid NHS ester was used to covalently attach PSI through reactions with lysine residues on the protein surface.^115^ Likewise, Ciornii et al. covalently immobilized PSI on multiwalled carbon nanotubes functionalized with carboxylated pyrene derivatives..^119^ Efrati et al. reported covalent assembly of PSI on pyrroloquinoline quinone (PQQ)-modified ITO electrodes using EDC coupling chemistry.^93^

Covalent immobilization has also been combined with nanoparticle integration in wild-type or engineered proteins. PSII isolated from *T. elongatus* was reconstituted with platinum nanoparticles (PtNPs) functionalized with 1-[15-(3,5,6-trimethyl-1,4-benzoquinone-2-yl)]­pentadecyl disulfide, which enabled incorporation of the PtNPs into the Q_B_ site of PSII. The PtNP-reconstituted PSII complexes were subsequently immobilized on Au(111) surfaces modified with 4,4′-biphenyldithiol.^150^ Zhu et al. engineered PSI by introducing six cysteine residues into the PsaE subunit, enabling covalent attachment to 4,4′-bis­(mercaptomethyl)­biphenyl-modified PtNP nanosheets supported on highly oriented pyrolytic graphite.^127^

Site-specific immobilization strategies have also been developed for bRCs. In one example, all native cysteine residues of the bRC were replaced with serine or alanine residues, after which two non-native cysteine residues were introduced into hydrophilic surface regions.^169^ This design enabled controlled and oriented immobilization of the bRC onto gold electrodes through stable Au–S linkages.

#### 3.3.3 3D Electrodes

A central strategy for improving the performance of biophotoelectrodes is to increase the loading of photosynthetic proteins on the electrode surface. This can be achieved by employing high-surface-area, three-dimensional electrode architectures (Figure 9d). Similar to conventional electrocatalysis, the structure and physicochemical properties of the electrode are engineered to maximize catalyst loading while simultaneously facilitating the mass transport of reactants and products and maintaining high electrical conductivity. Biophotoelectrochemical systems, however, impose an additional and often conflicting requirement: sufficient light must penetrate the electrode architecture to excite photosystems distributed throughout the porous matrix. This constraint limits the applicability of many classical opaque electrode materials, such as bulk metals and conventional carbon electrodes, and has motivated the development of specialized conductive materials that combine a high surface area with optical transparency.

Among these, ITO has emerged as the most widely used transparent material for constructing photosystem-based 3D electrodes. Stieger et al. employed mesoporous ITO electrodes to fabricate PSI-based photocathodes incorporating PSI from *Thermosynechococcus elongatus* together with horse-heart cytochrome *c*.^118^ Three-dimensional ITO electrodes can be prepared either from liquid precursor formulations or through nanoparticle-based fabrication routes, with precursor-derived electrodes generally providing superior optical transparency.^126^ Morlock et al. further demonstrated that mesoporous ITO architectures are suitable for direct electron transfer with PSI.^136^ High-surface-area ITO electrodes fabricated by electrospinning have also been developed for the PSI immobilization.^132^

ITO architectures additionally support PSI-based photoanodes employing dichlorophenolindophenol as electron mediator and ascorbate as sacrificial electron donor. In this context, macroporous ITO electrodes (∼5 μm pore size) generated photocurrent densities approximately 3-fold higher than mesoporous architectures with pore sizes of 20–100 nm.^128^ This improvement was attributed to enhanced accessibility for PSI multilayer formation and improved diffusion of redox mediators within the porous network.

Other transparent metal oxides have likewise proven to be effective for immobilization of photosynthetic proteins. Examples include porous antimony-doped tin oxide electrodes modified with bRCs from *Rba. sphaeroides*,^167^ fluorine-doped tin oxide (FTO) conducting glass used for electrodeposition of PSI,^129,137^ and nanostructured TiO_2_ electrodes used for electrospray deposition of PSI.^116^ The latter technique employs an electric field to direct charged droplets toward the electrode surface, enabling the controlled and homogeneous deposition of the proteins. PSI electrosprayed onto nanostructured TiO_2_ generated anodic photocurrent densities of up to 4.15 mA cm^–2^, representing one of the highest reported photocurrents for PSI-based photoelectrodes. In this case, however, the TiO_2_ substrate itself also contributes significantly to photocurrent generation.^116^

Other automated techniques such as airbrush spray-coating have also been applied to integrate PSI and PSII into a macroporous ITO electrode while simultaneously wiring the proteins using redox-active polymers.^130^ The scalability of this method was demonstrated through the production of electrodes with surface areas of up to 25 cm^2^.

Among the most successful architectures for achieving high PSII loadings are inverse opal mesoporous ITO (IO–mesoITO) electrodes, which possess hierarchical porous structures generated through templating approaches.^80,87,152^ When combined with redox-active polymers to electrically wire PSII throughout the 3D network, these electrodes produced photocurrent densities of up to ∼410 μA cm^–2^ (ref 80). Fang et al. systematically investigated structure–activity relationships in hierarchical PSII-based electrodes and found that architectures combining small macropores with mesopores larger than the dimensions of PSII enabled both higher enzyme loading and improved enzyme retention.^154^ Under DET conditions, however, the activity of individual PSII complexes was influenced more strongly by light intensity and electronic coupling at the bioelectrode interface than by electrode morphology alone. Inverse-opal mesoITO electrodes have also been successfully applied to RC–LH1-based photocathodes, yielding photocurrent densities as high as 4.6 mA cm^–2^ using cytochrome *c* as electron mediator.^170^

Three-dimensional carbon-based electrodes have also been investigated for photosynthetic protein immobilization. For example, PSII immobilized within macroporous carbon electrodes under both DET and MET conditions exhibited improved operational stability compared to porous ITO electrodes, although at the expense of lower photocurrent densities.^155^ This behavior was attributed to the limited optical transparency of carbon materials, which partially shielded PSII from photodamage. Notably, Morlock et al. demonstrated that reduced graphene oxide-based 3D electrodes can achieve partial optical transparency while supporting PSI immobilization, thereby providing a potentially more sustainable alternative to indium-based conductive materials.^133^

An alternative strategy for employing nontransparent materials in biophotoelectrodes involves exploiting plasmonic effects to enhance local light harvesting. Friebe et al. used nanostructured silver electrodes to construct RC–LH1 photoelectrodes from *Rba. sphaeroides*, achieving increased protein loading and enhanced photocurrent generation through plasmon-assisted light absorption.^97,99^ Similar plasmonic enhancement effects have been reported for PSI-based photoelectrodes employing silver island films, which increased both optical absorption and photocurrent generation under illumination.^141,187^

#### 3.3.4 Encapsulation in Conducting Film, Redox Polymers and Protein Matrix

An alternative approach for achieving high photosystem loading while remaining compatible with flat, nontransparent electrodes is the encapsulation of the photosynthetic proteins within electron conducting matrices. These matrices are typically composed either of intrinsically conducting π-conjugated polymers or of polymers functionalized with redox-active moieties capable of mediating long-range charge transport through electron-hopping mechanisms. Such conductive and redox-active materials simultaneously serve as immobilization scaffolds and electron-transfer mediators, thereby increasing photosystem loading while alleviating the need for control over protein orientation. These approaches have been widely applied to redox enzymes and have proven equally valuable for the fabrication of photosystem-based biophotoelectrodes.

Conducting polymers have attracted considerable interest because they can be readily synthesized by electropolymerization while exhibiting favorable electrical conductivity, optical transparency, and electrochemical stability. For example, PSI-based biophotoelectrodes were fabricated by sequential layer-by-layer deposition of PSI and the conducting polymer poly­(3,4-ethylenedioxythiophene):polystyrenesulfonate (PEDOT:PSS), with photocurrent increasing nearly linearly up to six deposited layers.^135^ Stachurski et al. further immobilized PSI on carbon-paper electrodes by coelectropolymerization with PEDOT:PSS.^138^ Similarly, Li et al. developed a PSII-based biohybrid photoanode by integrating PSII-enriched membranes within benzoquinone-doped polypyrrole films.^78^ The resulting benzoquinone-functionalized polypyrrole nanostructures combined a high surface area, excellent conductivity, and integrated redox mediation, leading to substantially enhanced photocurrent generation.

Redox-active polymers are particularly attractive because the redox potential of the pendant redox groups can be precisely matched to that of the photosystem, while the polymer backbone chemistry can be independently tailored to control solubility, interactions with the protein surface, and cross-linking chemistries^112^ (Figure 9e). Such polymers have been extensively employed for immobilization of PSII,^80,81,151^ PSI,^122,124,125,188,189^ and bRCs^98,190^ and have yielded some of the highest reported photocurrent densities, particularly when combined with three-dimensional electrode architectures.

Matching the redox potential of the polymer to that of the photosystem minimizes energetic losses associated with electron transfer between the protein and the electrode, thereby improving photocurrent generation and maximizing the open-circuit voltage of two-compartment systems combining PSII-based photoanodes with PSI-based photocathode.^79,191^ By optimizing electron-transfer kinetics within osmium-complex-modified polymer films encapsulating PSI, the rates of light-driven electron transfer achieved in PSI photocathodes were shown to exceed the electron-transfer rates observed in natural photosynthesis (Figure 5a).^89^ In these systems, photocurrent densities of up to 322 μA cm^–2^ were achieved using semiconductor-free electrodes, with further enhancement obtained through incorporation of SWCNTs into the Os-complex modified polymer matrix.^143^

The use of electron hopping within redox-active immobilization matrixes extends beyond synthetic polymers. For example, Stieger et al. constructed photoactive three-dimensional architectures by coassembling PSI and cytochrome *c* within nanoscale DNA-based matrices.^117^ In this system, the DNA scaffold together with cytochrome *c* formed stable layered assemblies that increased PSI loading, resulting in enhanced photocurrent generation and improved electrode stability.

### 3.4 Half-Cell Characterization

The rational design of the biophotoelectrochemical half-cells is essential for the development of high-performing devices. In classical bioelectrocatalysis, electroanalytical methods are routinely used because the measured current directly reflects the catalytic mechanism and its associated thermodynamic, kinetic, and mass transport parameters.^192^ In biophotoelectrochemical systems, however, the situation is fundamentally more complex. Multiple competing charge-recombination pathways, occurring between protein cofactors, electron donors, electron acceptors, and electrode interfaces, can partially or completely suppress the net photocurrent. As a result, the measured photocurrent alone does not enable us to deduce the rates of individual electron-transfer processes. Even when individual electron transfer steps are intrinsically fast, the overall photocurrent may remain low if recombination pathways dominate.

Disentangling the numerous interdependent electron-transfer processes occurring within photosystem-modified electrodes and identifying the associated kinetic bottlenecks and energetic losses, therefore, require a combination of complementary analytical approaches. In practice, this typically involves electrochemical characterization, product analysis, spectroscopic methods, and computational modeling.

#### 3.4.1 Electrochemical Methods

Electroanalytical techniques, particularly voltammetry and chronoamperometry, constitute the foundation for investigating photosystem-based biophotoelectrodes. When applied to photosystems immobilized directly on electrodes or embedded within thin films, these approaches are commonly referred to as *protein-film photoelectrochemistry*.^74,171^ In this technique, controlled illumination is combined with electrochemical potential control to probe photocurrent generation by photoactive proteins.

Chronoamperometry, in which the electrode potential is stepped while monitoring the current over time, is widely employed to optimize experimental conditions and evaluate photocurrent stability. The temporal evolution of the photocurrent provides valuable information on transport phenomena, recombination pathways, and degradation processes. For example, steady-state photocurrents may be obtained when the substrate is available in large excess, such as water for PSII-based photoanodes,^152^ and when the measurement time scale remains shorter than the time scale for protein deactivation. In contrast, Cottrell-type current decays (*j* ∝ *t* ^*‑*1/2^) is expected when soluble electron donors or acceptors become depleted during turnover, as observed for bRC-based systems operating with soluble Q_0_ under stationary conditions.^99^ The appearance of dark currents following illumination further indicates the presence of charge-recombination pathways. Beyond qualitative interpretation, the magnitude of the photocurrent itself provides a measure of the overall turnover rate of the photoinduced electron-transfer chain within the biophotoelectrode.^89^

Slow-scan cyclic voltammetry under alternating light and dark conditions is also routinely employed to determine photocurrent onset potentials and associated overpotentials (as discussed in section 3.2.2), thereby providing insight into the thermodynamic driving force required for productive charge extraction.

To directly detect products generated during biophotoelectrochemical reactions, techniques such as rotating ring–disk electrochemistry (RRDE) and scanning electrochemical microscopy (SECM) have proven particularly valuable. RRDE employs a dual-electrode geometry consisting of a central disk electrode surrounded by a concentric ring electrode. Products generated at the disk are transported to the ring electrode by forced convection induced through electrode rotation, where they can be electrochemically detected under independently controlled potentials. Using RRDE, Kornienko et al. investigated oxygenic photoreactivity in PSII-modified electrodes.^193^ Their study revealed that incomplete water oxidation was not the dominant source of reactive oxygen species (ROS). Instead, two distinct oxygen-reduction pathways were identified both involving a superoxide intermediate to produce H_2_O_2_. One pathway involved formation of singlet oxygen via chlorophyll triplet states followed by reduction to superoxide at the electrode surface. The second pathway originated at the enzyme–electrode interface, where exposed antenna chlorophylls accepted electrons directly from the electrode, generating strongly reducing chlorophyll anions capable of transferring electrons to dissolved O_2_ to produce superoxide directly.

SECM employs a microelectrode probe positioned close to the electrode surface to detect electroactive species generated or consumed locally, thereby enabling spatially resolved investigation of interfacial processes. Using SECM with a Pt microelectrode, hydrogen evolution from a PSI–Pt/hydrogel biophotoelectrode was directly detected under illumination.^188^ SECM has also proven highly valuable for probing charge-recombination kinetics. For example, the reduced methyl viologen radical commonly used as electron acceptor in PSI photocathodes is susceptible to reoxidation at the electrode surface, leading to recombination losses. SECM was used to quantitatively compare these recombination kinetics in PSI systems integrated with osmium-complex-modified polymers on gold and silicon electrodes.^189^ The study directly correlated photocurrent differences with the rate of methyl viologen reoxidation, revealing slower recombination kinetics on silicon electrodes and consequently lower recombination losses.

In another example, Zhao et al. used SECM to investigate degradation mechanisms in PSI/redox polymer biophotoelectrodes.^122^ Detection of H_2_O_2_ under illumination provided direct evidence that reactive oxygen species contributed significantly to electrode degradation.

Scanning photoelectrochemical microscopy (SPECM), an extension of SECM, combines localized illumination with electrochemical probing to simultaneously monitor photocurrent generation and chemical product formation at defined regions of the electrode surface. SPECM was employed to investigate electron-transfer pathways in PSII embedded within osmium-complex-modified redox polymers.^81^ Detection of H_2_O_2_ demonstrated the existence of competing oxygen-reduction pathways mediated by chlorophyll pigments. Furthermore, inhibitor studies targeting the Q_B_-plastoquinone site confirmed that electron transfer to the polymer occurred predominantly through the Q_A_ site.

#### 3.4.2 Spectroscopy and Spectroelectrochemistry

Spectroscopic methods, particularly when combined with electrochemical control, have proven to be exceptionally powerful for probing the structural integrity, electronic states, and kinetic bottlenecks of biophotoelectrodes.

A primary application of spectroscopy is verification of the structural and functional integrity of immobilized photosystems. For example, UV–Vis absorption spectroscopy is routinely used to confirm preservation of characteristic chromophore transitions, such as the Q_*y*_ and Soret bands of chlorophylls in PSI, thereby demonstrating that the protein remains structurally intact following immobilization.^129^ Absorption spectroscopy is also widely used to quantify protein loading on electrode surfaces,^89^ which is essential for determining turnover frequencies and internal quantum efficiencies.^99^

The contribution of photosystems to photocurrent generation is commonly verified by recording photocurrent action spectra, obtained by measuring photocurrent as a function of excitation wavelength.^91,98,100,145^ Agreement between the action spectrum and the optical absorption spectrum of the protein demonstrates that the immobilized photosystem is directly responsible for photocurrent generation (Figure 10).^100^

**Figure 10:**
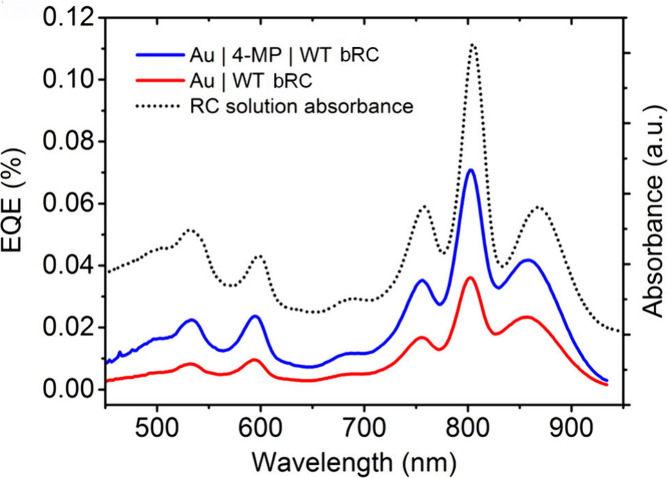
Comparison between the absorbance spectrum (dotted line) and the photocurrent action spectrum (solid line), expressed as the wavelength-dependent external quantum efficiency (EQE), of a bacterial reaction center-modified electrode. The close correspondence between both spectra confirms that the observed photocurrent originates from photoexcitation of the bRC complex. Reproduced with the permission from ref 100. Copyright 2016 Elsevier.

Spectroelectrochemical techniques enable direct monitoring of the redox states of key cofactors during operation. Using such approaches, Białek et al. investigated bRCs embedded within osmium-complex-modified hydrogels.^98^ By measuring absorption changes under controlled potentials, they determined the redox potentials of both the bRC primary donor and the osmium-complex mediator. Relative to bRCs in solution, incorporation into the hydrogel induced a positive shift in the donor redox potential, attributed to specific interactions between the protein and the polymer matrix. The resulting system achieved internal quantum efficiencies approaching 50%, indicating highly efficient electronic coupling between the bRCs and the redox polymer.^98^

Building upon these studies, Białek et al. employed transient absorption spectroscopy (TAS) to quantify electron-transfer kinetics between bRCs and redox polymers in solution.^190^ Analysis of the decay kinetics of the oxidized primary donor state (P^+^) revealed that electron-transfer rates strongly depended on the polymer-to-bRC ratio, with higher polymer concentrations accelerating charge transfer. These findings suggested that electrostatically driven complex formation between bRCs and redox polymers plays a central role in promoting efficient electron transfer.^190^

TAS has also been extensively applied to PSI-based biophotoelectrodes. Ultrafast TAS measurements were used to monitor the redox state of the PSI RC P700 under electrochemical potential control.^129^ Szewczyk et al. combined TAS with chronoamperometry to directly correlate intraprotein electron-transfer kinetics with photocurrent generation.^137^ Their study revealed that electrodeposition of PSI onto FTO electrodes resulted in severe loss of photoactivity, with up to 90% of immobilized PSI complexes becoming inactive. Partial recovery of activity could be achieved by applying sufficiently negative potentials or by adding chemical reducing agents. Furthermore, among the remaining photoactive PSI complexes, approximately half underwent rapid recombination through the P700^+^F_*x*_^–^ → P700F_*x*_ pathway before productive charge extraction could occur. These observations emphasize the importance of engineering interfaces capable of extracting and injecting electrons faster than intrinsic recombination processes within PSI.

Recent developments are extending spectroscopic analysis toward ultrafast multidimensional techniques. López-Ortiz et al. introduced photoelectrochemical two-dimensional electronic spectroscopy, which combines the ultrafast temporal resolution of two-dimensional electronic spectroscopy with simultaneous photoelectrochemical measurement.^194^ In principle, this approach enables direct correlation of energy-transfer dynamics, electronic coherences, and charge-separation pathways with photocurrent generation, thereby providing unprecedented mechanistic insight into the factors governing photoelectrode performance.

Spectroscopic methods are also essential for probing interactions between photosystems and artificial interfaces. Szalkowski et al. employed fluorescence spectroscopy to investigate energy transfer from photosynthetic complexes to monolayer graphene.^195^ Resonance-like features observed in the fluorescence spectra of both PSI and peridinin–chlorophyll *a* protein complexes were attributed to coupling with free carriers in graphene.

Similarly, Friebe et al. used *in situ* time-resolved spectroelectrochemistry to identify kinetic bottlenecks and loss pathways in bRC-based biohybrid photoelectrodes.^196^ Their analysis revealed two major inefficiencies: first, donor- and acceptor-side electron-transfer limitations rendered approximately 60% of immobilized bRCs inactive; second, parasitic recombination pathways between the charge-separated state and the electrode dissipated approximately 73% of the photogenerated charge from the remaining active bRCs. Such mechanistic insights provide a critical foundation for the rational design of next-generation, high-efficiency biophotoelectrodes.

#### 3.4.3 Computational Modeling and Simulation of Biophotoelectrodes

Electrochemical and spectroscopic characterization provides the experimental parameters required for computational modeling of biophotoelectrodes. Such models enable deconvolution of the multiple contributions underlying photocurrent generation, thereby providing a mechanistic understanding of electron-transfer pathways and predictive frameworks for device optimization.

Robinson et al. developed a reaction–diffusion model describing multilayer PSI films on electrode surfaces.^197^ The model treated the film as a three-dimensional matrix in which electroactive mediators diffuse while undergoing redox reactions coupled to light-driven charge separation within PSI. The resulting set of coupled partial differential equations successfully reproduced characteristic experimental observations, including sigmoidal catalytic voltammograms under illumination. More importantly, the simulations provided quantitative insight into spatial reaction distributions within the film, revealing that photocatalytic activity extended throughout the multilayer structure rather than being confined to the electrode interface. The model further demonstrated that photocurrent generation strongly depends on mediator diffusion coefficients and mediator concentration within the film.

Buesen et al. introduced a generalized electrochemical reaction–diffusion model for redox-active-film biophotoelectrodes that explicitly incorporates productive charge transfer and competing short-circuit pathways^198^ (Figure 11a). Extending established bioelectrochemical models^199,200^ the framework includes both light-driven electron transfer and recombination kinetics. The model predicts transient photocurrent responses while simultaneously resolving individual recombination contributions and concentration profiles of intermediates within the film. A particularly valuable feature of this approach is the derivation of dimensionless kinetic and transport parameters that allow straightforward identification of rate-limiting processes within the complex reaction network (Figure 11b). The model further enables simulation of how parameters such as charge-carrier diffusion coefficients influence photocurrent generation under different recombination regimes (Figure 11c). Such computational approaches are essential for bridging the gap between molecular-level reaction mechanisms and macroscopic device performance, thereby guiding the rational design of next-generation high-performance biophotoelectrodes.

**Figure 11:**
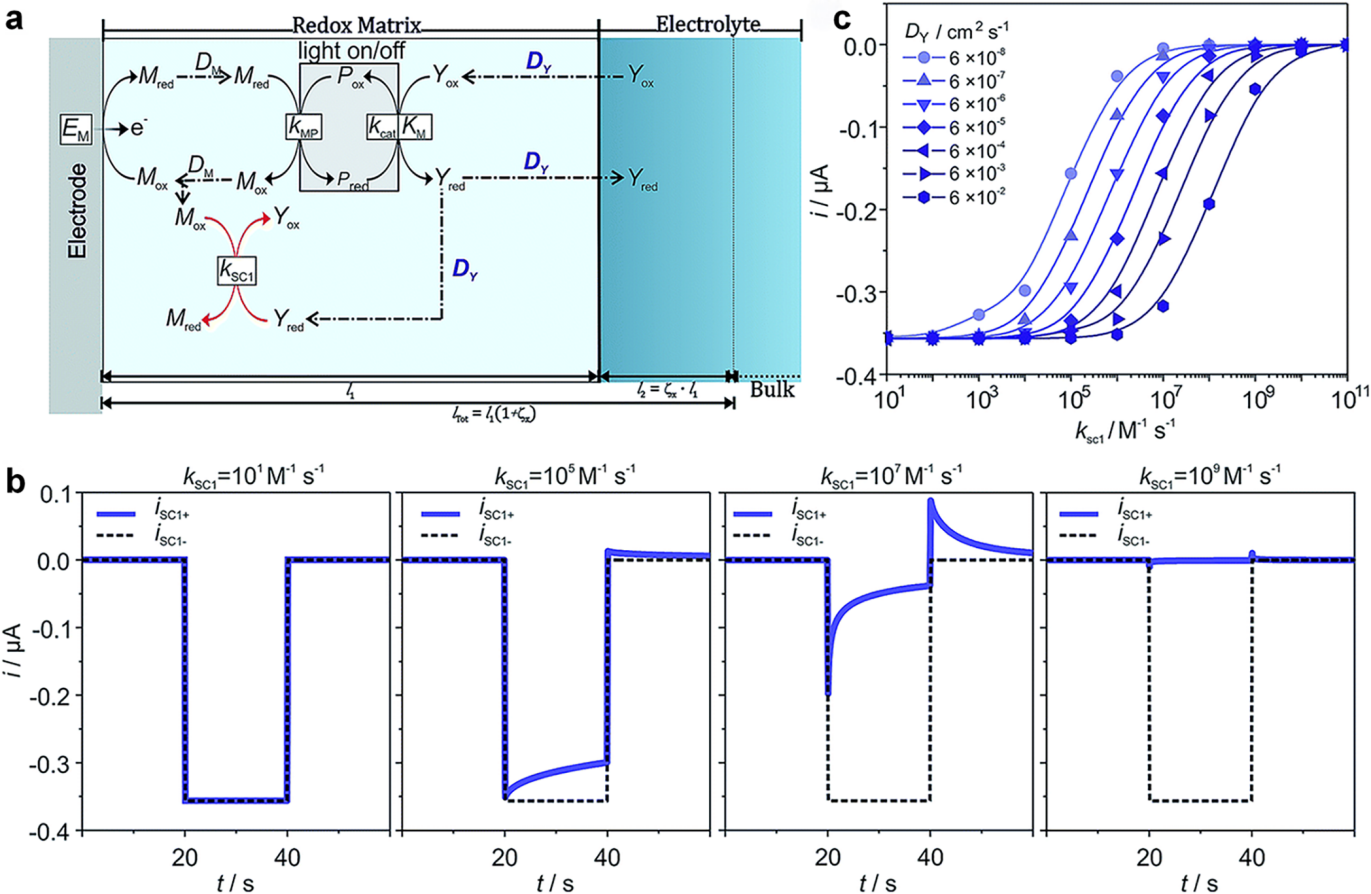
Electrochemical reaction–diffusion model describing charge transport and recombination processes in redox-active film-based biophotoelectrodes. (a) Schematic representation of the reaction network in a biophotocathode, where a photoactive protein generates a reduced charge carrier that diffuses into the bulk electrolyte. Charge recombination occurs through reaction of the reduced charge carrier with the oxidized electron mediator. (b) Simulated photocurrent response for the productive photocatalytic pathway alone (black dashed line) and including the competing recombination pathway (blue solid line) for increasing values of the short-circuit rate constant *k*_SC1_ at *D*_Y_ = 6 × 10^–6^ cm^2^ s^–1^. (c) Dependence of the photocurrent measured after 40 s of illumination on log­(*k*_SC1_) for different values of the diffusion coefficient *D*_Y_. Reproduced with the permission from ref 198. Copyright 2019 The Royal Society of Chemistry.

## 4 Applications of Biophotoelectrochemical Systems

Photosystem-based biophotoelectrodes have been integrated into a wide range of biophotoelectrochemical devices, enabling applications including light-driven electricity generation, full water splitting, H_2_ production, and CO_2_ reduction^31,201–203^ (Figure 12). For practical implementation, such systems must operate without sacrificial electron donors or acceptors, while solar fuel-producing devices should ideally function under bias-free conditions. Achieving these objectives generally requires complementary engineering of both the photoelectrode and counter electrode to ensure efficient closure of the electrochemical circuit and suppression of charge recombination pathways. This section reviews recent advances in photosystem-based photoelectrochemical cells that address these challenges.

### 4.1 Electricity Generation

The integration of photosystems into photovoltaic devices aims to convert light directly into electrical energy (Figure 12a). In these systems, photoexcitation of the immobilized photosystem generates charge carriers that are collected at the counter-electrode to produce an electrical current. Charge transport between the two electrodes can proceed through freely diffusing redox mediators, analogous to DSSC, through immobilized redox carriers in solid-state electrolytes or via direct electrical wiring in fully solid-state biophotovoltaic architectures. Most reported devices combine photosystems with conventional photoelectrode materials, including metal oxides, perovskites, and *n*-doped silicon, which often contribute substantially to the overall light-to-electricity conversion. Table 1 summarizes the performance of representative biophotovoltaic systems employing different device configurations.

Current–voltage (*I*–*V*) characterization constitutes the primary method for evaluating the performance of complete biophotovoltaic cells. In these measurements, the current response is recorded as a function of the applied voltage under illumination, typically together with measurements performed in the dark as a control. From the resulting *I*–*V* curves, the short-circuit current density (*J*_SC_) is determined at zero applied voltage, while the open-circuit voltage corresponds to the voltage at which the current becomes zero. The overall light-to-electricity conversion efficiency (η) can then be calculated as described in section 3.2.4. In addition, quantum efficiencies are commonly determined under short-circuit conditions. The EQE and IQE respectively quantify the conversion efficiencies of incident photons and absorbed photons into electrical current.

#### 4.1.1 DSSC-Type Architectures with Liquid Electrolytes

The implementation of PSI in DSSC-type biophotovoltaic devices has thus far focused predominantly on PSI functioning as a photoanode in combination with soluble redox mediators. In these systems, conventional DSSC redox couples, such as Co­(II)/Co­(III)-based complexes or the I^–^/I_3_^–^ couple, serve as charge carriers. The primary objective of these architectures has generally been to improve the sustainability and potentially reduce the cost of existing DSSC technologies, with PSI acting mainly as a molecular photosensitizer and an interfacial charge-transfer catalyst rather than as the sole photovoltaic component.

**Figure 12:**
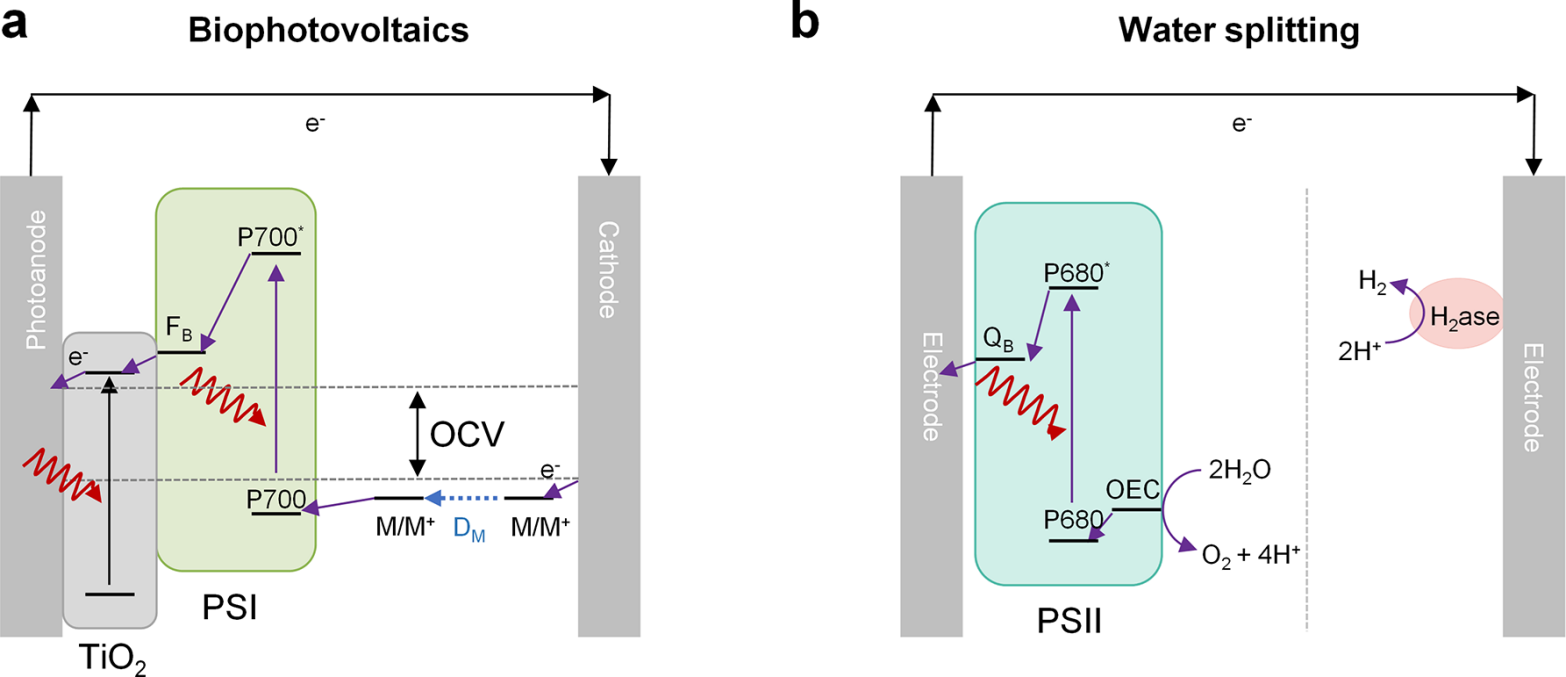
Representative applications of PSI- and PSII-based systems for solar-energy conversion. (a) PSI-based biophotovoltaic device operating in a dye-sensitized solar cell (DSSC)-type configuration. (b) Light-driven overall water splitting achieved by coupling a PSII-based photoanode to a hydrogenase-modified cathode for photocatalytic H_2_ evolution.

Mershin et al. assembled PSI on mesoporous TiO_2_ or ZnO electrodes to fabricate DSSC-type PSI biophotovoltaic devices.^95^ These systems achieved an *V*_oc_ of approximately 0.5 V and a photocurrent density of 362 μA cm^–2^. Yu et al. immobilized PSI from *Arthrospira platensis* and spinach light-harvesting complex II (LHCII) on TiO_2_ electrodes and systematically investigated the influence of TiO_2_ particle size and pore architecture.^205^ The PSI-based device achieved a short-circuit current of 1.31 mA cm^–2^ and an energy conversion efficiency of 0.47%, while the LHCII-based devices reached 1.51 mA cm^–2^ and 0.52%. Takekuma et al. further reported a tandem biophotovoltaic cell combining a NiO/PSI photocathode with a TiO_2_/PSI photoanode, achieving an *V*_OC_ of 0.7 V using the I^–^/I_3_^–^ redox couple as the electrolyte.^209^

Hybrid sensitization strategies have also been explored to broaden light absorption. In one example, PSI multilayers were deposited onto mesoporous TiO_2_ films presensitized with natural anthocyanin dyes, while the [Fe­(CN)_6_]^4–^^/^^3–^ redox couple served as the electrolyte mediator.^96^ Incorporation of PSI enhanced overall light harvesting through the complementary absorption profiles of PSI and the anthocyanin dye, resulting in approximately 2-fold higher photovoltages relative to dye-only devices. Teodor et al. later investigated synthetic bipyridine-based cobalt mediators soluble in either aqueous or organic solvents for PSI-based DSSCs.^224^ The aqueous cobalt complex enabled a 38-fold enhancement in PSI reduction kinetics compared with the analogous organic-soluble mediator. Building on this concept, Teodor et al. subsequently developed PSI-based DSSCs employing PEDOT/CNT counter electrodes together with water-soluble cobalt bipyridine mediators to improve biocompatibility and charge-transfer efficiency at the biological–inorganic interface.^211^

Although PSI-based photocathodes can achieve photocurrent densities in the mA cm^–2^ range, they have only rarely been integrated into complete biophotovoltaic cells because of their strong susceptibility to charge recombination. In most PSI photocathode systems, recombination losses are mitigated through the use of sacrificial electron acceptors. However, these irreversible reactions are fundamentally incompatible with biophotovoltaic architectures that require reversible charge-carrier cycling between both electrodes.

In contrast, photocathodes based on purple bRCs, which exhibit intrinsically lower recombination rates, have been more successfully integrated into DSSC-type devices. For example, an FTO/RC–LH1 photocathode coupled to an *n*-doped silicon anode achieved an *V*_oc_ of 0.7 V using 2,2,6,6-tetramethyl-1-piperidinyloxy (TEMPO) as a redox mediator.^207^ RC–LH1 photoanodes have also been combined with PEDOT:PSS cathodes using the Q_0_/Q_0_H_2_ couple as charge carrier.^206^ However, these systems generated only small open-circuit voltages (∼4 mV), indicating inefficient cycling of charge carriers between the two electrodes. Yaghoubi et al. assembled bRCs on ZnO nanowire electrodes and employed either ferrocene or methyl viologen as electrolyte mediators.^208^ Incorporation of ZnO partially suppressed charge recombination. Using methyl viologen yielded photocurrent densities of 5.5 μA cm^–2^ with *V*_oc_ values of 36 mV, while ferrocene produced slightly higher values of 6.4 μA cm^–2^ and 41 mV, respectively.

PSII-based photoanodes have likewise been incorporated into DSSC architectures using the I^–^/I_3_^–^ redox couple achieving *V*_oc_ of 0.495 V together with short-circuit current densities of 888 μA cm^–2^ (ref^210^). Nevertheless, the practical implementation of PSII-based devices remains severely limited by the intrinsically low operational stability of PSII under continuous illumination and catalytic turnover.

**Figure 13:**
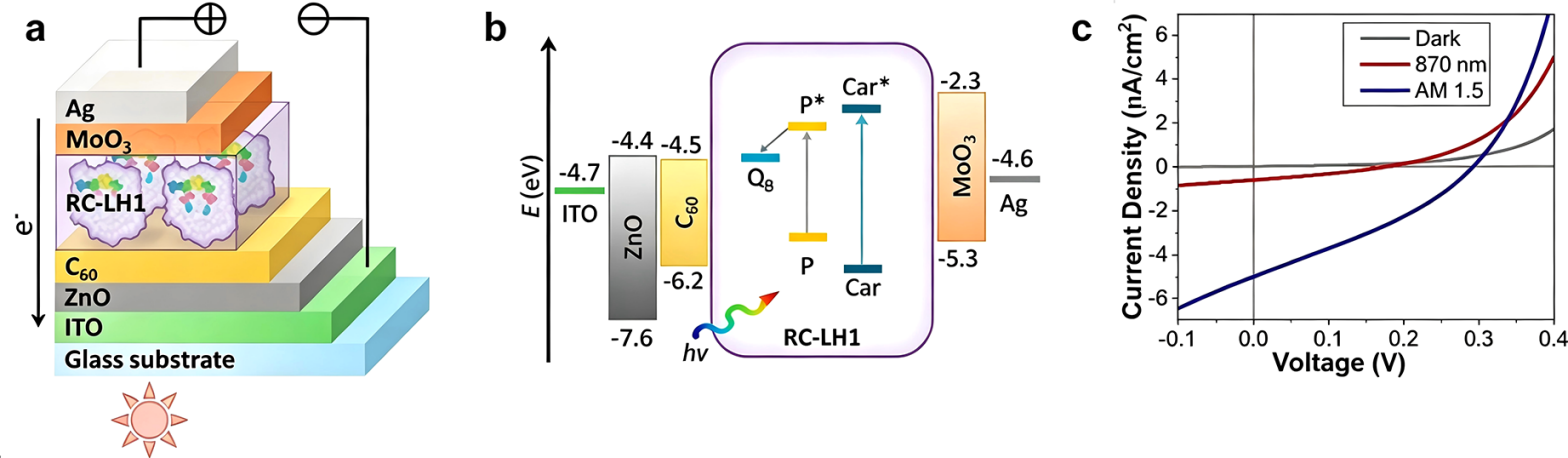
Solid-state biophotovoltaic device based on a layered ITO/ZnO/C_6__0_//RC-LH1/MoO_3_/Ag architecture. (a) Schematic representation of the device structure and the direction of light-driven electron transfer. (b) Energy-level diagram illustrating charge separation and carrier transport within the device. (c) Current density–voltage (*J*–*V*) characteristics of the ITO/ZnO/C_60_/RC-LH1/MoO_3_/Ag biophotovoltaic cell. Reproduced with the permission from ref 220. Copyright 2025 Wiley.

#### 4.1.2 DSSC-Type Architectures with Solid-State Electrolytes

The use of liquid electrolytes containing mediators such as methyl viologen introduces several major challenges for long-term device operation including electrolyte leakage, electrode corrosion, and enhanced charge recombination. To overcome these limitations and improve scalability and operational stability, solid-state approaches for PSI-based photovoltaics have been explored. In one example, polyviologen was employed as a solid-state electrolyte in PSI-based DSSCs.^212^ Compared with analogous systems employing dissolved methyl viologen mediators, the resulting solid-state devices exhibited substantially higher photocurrents, demonstrating the promise of immobilized charge carrier architectures for stable biophotovoltaic operation.

**Table 1:** Performances of Representative Biophotovoltaic Devices

		electrode							
ref and years	protein	anode	cathode	charge carrier	*V*_oc_ (V)	*J*_sc_ (μA cm^–2^)	*ff*	conversion efficiency η (%)	illumination
DSSC, Liquid Electrolyte									
2012 ^95^	PSI	TiO_2_/PSI	Pt	Co(II)/Co(III)	0.5	362	0.6	0.08	AM1.5G
2014 ^204^	PSI	Fe_2_O_3_/PSI	Pt	I^–^/I_3_^–^	0.321	56.9	0.56	0.17	AM1.5G
2015 ^205^	PSI	TiO_2_/PSI	Pt	I^–^/I_3_^–^	0.59	1310	0.62	0.47	AM1.5G
2017 ^206^	RC-LH1	PEDO:PSS	FTO/RC-LH1	Q_0_/Q_0_H_2_	0.004	8.6			AM1.5G
2017 ^207^	RC-LH1	n-doped Si	FTO/RC-LH1	TEMPO	0.7	8.2		0.00575	white light 100 mW cm^–2^
2017 ^208^	bRC	Pt	FTO/ZnO cyt c/bRC	ferrocene	0.041	6.4			AM 1.0 80 mW cm^–2^
2018	PSI	TiO_2_/PSI	Pt	Fe(CN)_6_^4–/3–^	0.5	80			380–790 nm 140 mW cm^–2^
2020 ^209^	PSI	TiO_2_/PSI	NiO/PSI	I^–^/I_3_^–^	0.71	160	0.5	0.06	AM1.5G
2020 ^210^	PSII	TiO_2_/PSII	Pt	I^–^/I_3_^–^	0.495	888	0.5	0.22	AM1.5G
2022 ^211^	PSI	TiO_2_/PSI	PEDOT/CNT	Co(II)/Co(III)	0.259	11.25	0.24	0.7	
DSSC, Solid-State Electrolyte									
2018 ^212^	PSI	ITO	p-Si/PSI	polyviologen	0.25	27		0.002	AM1.5G

ref and years	protein	electrode			*V*_oc_ (V)	*J*_sc_ (μA cm^–2^)	*ff*	conversion efficiency η (%)	illumination
All Solid-State Biophotovoltaics									
2004 ^213^	bRC	Au/bRC/C_60_/BCP/Ag			0.025	120			808 nm 10^4^ mW cm^–2^
2014 ^214^	PSI	ITO/TiO_*x*_/PSI/PTAA/MoO_3_/Al			0.76	0.29	0.45	0.006	AM1.5G > 435 nm
2015 ^215^	PSI	p-doped Si/PSI/ZnO/ITO			0.214	127	0.28	0.008	AM1.5G
2015 ^216^	PSI	FTO/TiO_2N_/Pann-PSI/Ag			0.3	72	0.42	0.0091	AM1.5G
2017 ^217^	PSI	FTO/PEDOT: PSS/PSI/LiF/Al			0.25	960	0.31	0.069	AM1.5G
2021 ^218^	PSI	ITO/PSI/Au			0.172	4000	0.25	0.22	AM1.5G
2022 ^219^	PSI	ITO/PY/PANIITO/PY/Pani/PSI/rGO-Au/Au			0.3	5600	0.38	0.64	AM1.5G
2025 ^220^	RC-LH1	ITO/ZnO/C_60_/RC-LH1/MoO_3_/Ag			0.3	5.1	0.32	0.0005	AM1.5G
Perovskite Solar Cell									
2023 ^221^	PSI	FTO/TiO_2_/Perovskite/PSI/Spiro-OMeTAD/Au			1.06	23.41 × 10^3^	0.76	18.63	AM1.5G
2024 ^222^	PSI	FTO/TiO_2_/Perovskite/PSI/CNT/Spiro-OMeTAD/Au			1.08	23.24 × 10^3^	0.77	18.95	AM1.5G

ref and years	protein	anode	cathode	charge carrier	*V*_oc_ (V)	*J*_sc_ (μA cm^–2^)	*ff*	conversion efficiency η (%)	illumination
Two Biohybrid Electrodes Photovoltaics									
2019 ^223^	PSII	IO-TiO_2_/PbS QD/P_Os_/PSII	IO-ATO/BOD	H_2_O/O_2_	1	76	0.60		white light 100 mW cm^–2^
2021 ^94^	PSI	Au/P_vio_/PSI/P_Os_/GOx	GOx/HRP/carbon		0.96	7.2			white light 113 mW cm^–2^

#### 4.1.3 Fully Solid-State Biophotovoltaic Devices

A distinct class of fully solid-state biophotovoltaic devices operates entirely without diffusible charge carriers by directly interfacing both sides of the PSI with solid electrodes. The major challenge in these systems lies in maintaining the structural integrity and photoactivity of the photosystem while simultaneously achieving preferential orientation and efficient electronic coupling between PSI and both electrodes. Because both interfaces are intrinsically interconnected, these systems cannot be optimized independently as half cells and instead must be assembled and characterized directly as complete devices.

Beam et al. fabricated a fully solid-state biophotovoltaic device with a layered p-Si/PSI/ZnO/ITO architecture by depositing crystalline ZnO directly onto PSI films.^215^ Habibi et al. developed mediator-free PSI devices by orienting PSI complexes using externally applied pulsed electric fields, resulting in approximately 4-fold enhancement in device efficiency.^218^ Gizzie et al. fabricated PSI-based solid-state biophotovoltaics by coelectropolymerizing PSI with polyaniline (PAni) on TiO_2_ anode.^216^ These devices generated photocurrent densities of 72 μA cm^2^ and open-circuit voltages of 300 mV. Kazemzadeh et al. constructed layered solid-state devices by depositing PSI multilayers (∼800 nm thick) onto PEDOT:PSS conductive films and completing the architecture with LiF/Al top electrode.^217^ Torabi et al. further developed reduced graphene oxide/gold nanoparticle electron-transfer layers for PSI-based solid-state photovoltaics.^219^ Their optimized configuration achieved external power-conversion efficiencies of 0.64% using PSI as the sole photoactive component. Finally, solid-state devices with layered ITO/ZnO/C_6__0_//RC-LH1/MoO_3_/Ag architectures achieved *V*_oc_ of approximately 0.3 V, representing the highest reported voltages for fully solid-state solar cells based on RC–LH1 complexes (Figure 13).^220^

PSI has also been incorporated into perovskite solar cells as a strategy to enhance photovoltaic performance. He et al. integrated PSI into lead-halide perovskite solar cells, exploiting the complementary absorption profiles of PSI and the perovskite absorber layer. This integration enhanced the external quantum efficiency and increased the overall power-conversion efficiency from 17.84% to 18.63% (ref 221). Zhang et al. further improved PSI–perovskite devices by introducing a carbon nanotube interfacial layer, resulting in simultaneous enhancements in both the open-circuit voltage and the external quantum efficiency.^222^

#### 4.1.4 Biophotovoltaic Devices Based on Two Biohybrid Electrodes

PSII- and PSI-based photoelectrodes have also been coupled with enzyme-modified counter electrodes to construct complete biophotovoltaic cells for electricity generation. An *V*_oc_ of approximately 1 V was achieved using a TiO_2_ inverse-opal photoanode functionalized with PSII and PbS quantum dots for light-driven water oxidation, combined with an inverse-opal antimony-doped tin oxide cathode modified with bilirubin oxidase to catalyze oxygen reduction.^223^ Wang et al. fabricated a PSI-based photoanode by depositing a PSI monolayer via the Langmuir–Blodgett technique and electrically wiring the protein with redox polymers.^94^ This photoanode was integrated into a biophotovoltaic cell containing an air-breathing biocathode functionalized with glucose oxidase and horseradish peroxidase. Under illumination, the device generated an open-circuit voltage of approximately 960 mV, in close agreement with the theoretical potential difference expected from the two coupled bioelectrode reactions.

### 4.2 Solar Fuels Production

#### 4.2.1 H_2_ Generation

Although PSI-based photocathodes have not yet achieved widespread implementation in biophotovoltaic systems for electricity generation, they remain the only photosystem-based photoelectrodes capable of driving light-induced H_2_ production.^225,226^ This unique capability originates from the highly negative reduction potential of the terminal [4Fe–4S] clusters (F_A_/F_B_), from which electrons are extracted from PSI (Figure 2f). In contrast, the natural electron-exit sites of PSII and bRCs (Q_B_ sites) are several hundred millivolts too positive to thermodynamically support proton reduction.

The operating principle of PSI-based photocathodes for hydrogen evolution relies on the photoexcitation of PSI to generate highly reducing electrons that are subsequently transferred to proton-reduction catalysts, such as platinum nanoparticles (Pt) or hydrogenases (H_2_ases), which catalyze the formation of molecular hydrogen. The reported performances of PSI-based photocathodes and PSI-Pt/H_2_ase assemblies for light-driven H_2_ production are summarized in Table 2.

**Table 2:** Performance of PSI-Based Biohybrid Systems for Light-Driven H_2_ Generation

ref and years	protein complex	products	electron donor	reaction rate	photocurrent (μA cm^–2^)
2006 ^227^	PSI-H_2_ase complex	H_2_	Asc/TMPD	0.58 μmol H_2_· mg chlorophyll^–1^· h^–1^	
2008 ^228^	PSI-Pt	H_2_	Asc/DCPIP	49.3 μmol H_2_· mg chlorophyll^–1^· h^–1^	
2009 ^229^	PSI-H_2_ase complex	H_2_	electrode	120 ± 30 pmol H_2_ s^–1^ cm^–2^	0.085
2009 ^230^	PSI-Pt	H_2_	Asc/DCPIP	312 μmol H_2_· mg chlorophyll^–1^· h^–1^	
2015 ^188^	PSI-Pt	H_2_	electrode		4.8
2017 ^90^	PSI, H_2_ase	H_2_	electrode		30
2019 ^231^	PSI-Pt	H_2_	Asc/DCPIP	26.3 mol H_2_ (mol PSI) ^–1^ h^–1^	

Ihara et al. engineered a fusion protein consisting of a membrane-bound [NiFe]-hydrogenase from *Ralstonia eutropha* H16 and the peripheral PSI subunit PsaE from *Thermosynechococcus elongatus*.^227^ The resulting PsaE–hydrogenase fusion protein spontaneously assembled with PsaE-deficient PSI to form a functional PSI–hydrogenase biohybrid complex. In the presence of ascorbate/*N*,*N*,*N*′,*N*′-tetramethyl-*p*-phenylenediamine (TMPD) as sacrificial electron donor, the system exhibited light-driven hydrogen evolution at a rate of 0.58 μmol H_2_ mg chlorophyll^–1^ h^–1^.

Building on this concept, Krassen et al. directly fused the same membrane-bound [NiFe]-hydrogenase from *R. eutropha* H16 to PSI from Synechocystis sp. PCC 6803.^229^ Introduction of a decahistidine tag enabled immobilization of the PSI–hydrogenase hybrid complex onto Ni–NTA-modified gold electrodes. Under illumination, the immobilized assemblies generated hydrogen at rates of 4500 ± 1125 μmol H_2_ min^–1^ mol^–1^ of biohybrid complex. A major advantage of such PSI-coupled systems is that the photogenerated reducing power of PSI enables hydrogen evolution at substantially more positive electrode potentials, and therefore lower energetic cost, than conventional Pt-based cathodes.

PSI has also been covalently linked to Pt and Au nanoparticles using 1,6-hexanedithiol molecular wires, enabling electron transfer from the terminal F_B_ cluster of PSI to the nanoparticle catalyst for proton reduction.^228^ Grimme et al. subsequently investigated the influence of pH, ionic strength, electron-donor mobility, and molecular-wire structure on the performance of PSI–Pt conjugates.^230^ Among the different linkers examined, 1,4-benzenedithiol yielded the highest hydrogen-production activity, reaching rates of 312 mmol H_2_ mg Chl^–1^ h^–1^. In a subsequent study, electrostatically assembled PSI–Pt complexes were incorporated into redox hydrogels to construct biophotocathodes for photoelectrocatalytic hydrogen evolution at low overpotential.^188^ Furthermore, Nakagawa et al. demonstrated that incorporation of the artificial light-harvesting dye Lumogen Red into electrostatically assembled PSI–Pt complexes enhanced light-driven hydrogen production efficiency.^231^

Light-driven hydrogen generation using PSI and hydrogenases can also be achieved without direct fusion of the two proteins through hierarchical layer-by-layer electrode architectures.^90^ In this approach, an Os-complex-modified redox polymer was first deposited onto the electrode surface, followed by a PSI layer and finally a second redox polymer film containing hydrogenase. This compartmentalized assembly promotes directional electron transfer and minimizes charge recombination, thereby enabling efficient transfer of photogenerated electrons from PSI to the hydrogenase catalyst for proton reduction. The resulting photocathodes exhibited photocurrents with onset potentials as positive as +0.38 V vs SHE, and the photocurrent magnitude correlated directly with the rate of hydrogen evolution, confirming productive light-driven catalysis.

#### 4.2.2 Full Water Splitting

PSII is the only known enzyme capable of catalyzing light-driven water oxidation. Coupling a PSII-based photoanode with a cathode capable of proton reduction therefore enables, in principle, complete water splitting into molecular oxygen and hydrogen (Figure 12b). However, because PSII alone does not generate sufficiently negative reducing potentials to drive proton reduction directly (Figure 2f), additional energy input is required. This additional driving force can be supplied either through an external electrical bias or through a second photoexcitation step in a Z-scheme-type architecture employing semiconductor photoelectrodes, organic photosensitizers, or PSI-based photocathodes.

Although the limited operational stability of PSII currently prevents its practical implementation in solar-fuel devices, PSII-based systems remain highly valuable model platforms. They provide important mechanistic and engineering insights into the integration of water oxidation catalysts within biophotoelectrochemical architectures and guide the development of more robust artificial and hybrid water-splitting systems. A summary of reported photosystem-based biophotoelectrochemical systems for full water-splitting is presented in Table 3.

**Table 3:** Photosystem-Based Biophotoelectrochemical Systems for Full Water Splitting

ref and years	anode	cathode	reaction rate	*J*_photo_ (μA cm^–2^)	*V*_Bias_ (V)	solar-to-hydrogen efficiency (%)	illumination
2015 ^148^	ITO/PSII	ITO/H_2_ase	O_2_: 0.52 μmol h^–1^ cm^–2^	450	0.9	1.5	660 nm 10 mW cm^–2^
			H_2_: 0.96 μmol h^–1^ cm^–2^				

2016 ^232^	PSII	Pt-decorated Si	O_2_: 15 μmol h^–1^		0	0.29	>420 nm 250 mW cm^–2^
			H_2_: 30 μmol h^–1^				

2017 ^233^	PSII, Pt	CdS	O_2_: 8.5 μmol h^–1^	120	0	0.34	AM1.5G
			H_2_: 17.7 μmol h^–1^				

2018 ^234^	ITO/PSII	Si/TiO_2_/H_2_ase	H_2_: 0.23 μmol h^–1^ cm^–2^		0.4		AM1.5G 100 mW cm^–2^ > 420 nm
2018 ^235^	FTO/TiO_2_/dpp/P_Os_-PSII	FTO/ITO/H_2_ase	H_2_: 0.06 μmol h^–1^ cm^–2^	28	0	0.14@0.3 V	AM1.5G 100 mW cm^–2^ > 420 nm

2019 ^125^	Au/P_Os_/PSII	Au/P_Os_/PSI/P_vio_/H_2_ase		0.52	0		anode: > 600 nm 32 mW cm^–2^
							cathode: white light 246 mW cm^–2^

Early photosystem-based systems for complete water splitting relied on the application of an external electrical bias to overcome the insufficient thermodynamic driving force between the photoanode and cathode reactions. Mersch et al. developed a photoelectrochemical cell combining a PSII-based photoanode with a hydrogenase-modified cathode for light-driven water splitting.^148^ Hierarchically structured ITO electrodes were employed on both sides of the cell to achieve high loadings of both PSII and hydrogenase catalysts. However, because of the energetic mismatch between the redox potential of the terminal quinone acceptor of PSII and the iron–sulfur clusters of the hydrogenase, an additional external bias greater than 0.6 V was required to drive sustained hydrogen evolution. This bias requirement was subsequently reduced to approximately 0.4 V by coupling the hydrogenase catalyst to a *p*-type silicon inverse-opal TiO_2_ photocathode, thereby introducing an additional photovoltage contribution from the semiconductor electrode.^234^

Bias-free operation has also been demonstrated by integrating PSII into a diketopyrrolopyrrole (dpp)-sensitized TiO_2_ photoanode and coupling it with a hydrogenase cathode.^235^ Additional bias-free approaches include tandem architectures composed of a PSII photoanode paired with a Pt-decorated silicon photocathode,^232^ CdS-PSII hybrid photoelectrochemical cell^233^ and systems that couple a PSI-hydrogenase photocathode with a PSII photoanode.^125^ In the latter configuration, the photocathode was fabricated using Langmuir–Blodgett deposition to form a densely packed and anisotropically oriented PSI monolayer. This controlled orientation minimizes charge recombination and promotes efficient directional electron transfer to the hydrogenase for proton reduction. When coupled with a PSII photoanode, the resulting tandem system achieves overall water splitting driven exclusively by light.

#### 4.2.3 CO_2_ Reduction and Other Applications

The highly negative reduction potential of the terminal [4Fe–4S] cluster F_B_ in PSI is also sufficient to drive CO_2_-reduction reactions.^1000^ Exploiting this property, Morlock et al. integrated PSI and formate dehydrogenase (FDH) within inverse-opal ITO electrode to construct a biophotocathode for the reduction of CO_2_ to formate.^236^ The resulting ITO/PSI/FDH half-cell achieved a Faradaic efficiency of 15% at an applied potential of −0.2 V vs Ag/AgCl.

In a related approach, a full biophotoelectrochemical cell for CO_2_ reduction was assembled by combining a PSII-based photoanode, incorporated into a dpp-sensitized TiO_2_ electrode, with a tungsten-dependent FDH cathode. This tandem configuration enabled the conversion of CO_2_ to formate with a minimal applied bias of only 0.3 V.^237^ Lian et al. reported another full photoelectrochemical system for CO_2_ reduction based on a PSII photoanode paired with a *p*-type silicon nanowire (Si-NW) photocathode, achieving the reduction of CO_2_ to methanol under an applied bias of 0.5 V.^238^

Beyond applications in CO_2_ reduction, PSI has been reported to be conjugated to ferredoxin and coupled with FNR for the assembly of a photocathode for light-driven NADPH generation.^239^

## 5 Advancing Performance toward Practical Deployment

Despite major advances in photocurrent generation over the past decade, the practical deployment of photosystem-based biophotoelectrodes remains constrained by several fundamental limitations. In contrast to microbial biophotoelectrochemical systems,^240^ which benefit from intrinsic cellular self-repair, metabolic cofactor regeneration, and long-term viability, platforms that rely on isolated photosystem-based electrodes often display reduced operational stability. Additional challenges arise from the relatively narrow spectral absorption range of individual photosynthetic proteins and from interfacial losses associated with their immobilization on electrodes, including an unfavorable orientation, incomplete wiring, and charge recombination. Together, these factors currently limit both the achievable power output and the long-term durability of subcellular biophotoelectrochemical devices.

Nevertheless, photosystem-based systems also offer important advantages over microbial platforms, including higher photocurrent densities, more defined electron-transfer pathways, and superior tunability through protein engineering. The absence of competing metabolic pathways enables more direct extraction of photogenerated electrons, while the simplified and highly controllable bio–abiotic interface facilitates mechanistic investigation, targeted protein engineering, and rational optimization of electron-transfer pathways. Furthermore, isolated protein architectures are generally more compatible with integration into nanostructured electrodes, redox-active polymers, and semiartificial photoelectrochemical devices. Recent advances in protein engineering, materials integration, and electrode design are therefore beginning to address key bottlenecks associated with stability and interfacial charge transfer, opening promising routes toward practical biophotoelectrochemical applications.

### 5.1 Protein Engineering

The performance of the biophotoelectrochemical systems is often limited by the inherent properties of native photosynthetic proteins, which evolved for operation within biological membranes rather than on abiotic electrochemical interfaces. Recent advances in protein engineering, ranging from targeted mutagenesis of natural complexes to the *de novo* design of artificial photoactive proteins, provide powerful opportunities to overcome these limitations and develop highly efficient, application-specific biophotoelectrochemical systems.

One promising strategy involves the targeted redesign of natural photosynthetic complexes to enhance or redirect electron transfer pathways. Fu et al. engineered a novel electron-transfer pathway in PSII from *Chlamydomonas reinhardtii* by modifying the Q_A_ site.^241^ Using 2,6-dimethyl-*p*-benzoquinone (DMBQ) as an electron mediator, they demonstrated that specific mutations, particularly truncation of the C-terminus of the PsbT subunit, shortened the electron-transfer distance between Q_A_ and DMBQ and significantly enhanced mediator reduction rates. Such strategies are directly relevant to biophotoelectrochemistry because they provide routes to engineer photosystems with improved electronic communication with external mediators and electrode surfaces, thereby increasing the level of photocurrent generation. This concept was further explored by Hartmann et al., who investigated electron-transfer properties of natural variants of the PSII D1 subunit (PsbA) using a redox-polymer-based biophotoelectrochemical platform.^151^ Their work demonstrated that even naturally occurring sequence variations can substantially influence electron-transfer kinetics, suggesting that engineered PsbA variants could be optimized for specific electrochemical environments.

Beyond modifying natural proteins, an even more transformative direction involves the *de novo* design of artificial photoactive proteins, which are often termed maquettes. These minimalist proteins are constructed from first-principles to perform specific light-driven electron-transfer functions without the structural complexity and evolutionary constraints of natural photosystems. Through rational design, Ennist et al. developed a reaction-center maquette and established computational methodologies for designing proteins capable of binding cofactors such as chlorophylls and quinones in precisely defined geometries to promote charge separation.^242^ The ability to engineer systems with tunable redox potentials, optimized electron transfer pathways, and simplified architectures opens exciting opportunities for the development of fully artificial photosynthetic systems for solar-fuel generation.

Building upon this work, Ennist et al. subsequently demonstrated the *de novo* design of synthetic photochemical reaction centers capable of efficient light-induced charge separation.^243^ This achievement represents a major conceptual breakthrough, showing that entirely artificial proteins can not only structurally resemble natural RCs but also reproduce their core photochemical functions. Such systems drastically expand the design space for biophotoelectrochemistry because researchers are no longer restricted to naturally evolved proteins. Instead, fully synthetic biophotocatalysts with tailored stability, electrode-binding properties, solvent compatibility, and redox characteristics can, in principle, be designed specifically for targeted photoelectrochemical applications.

### 5.2 Extending the Absorption Range

A major limitation of native photosynthetic proteins is their relatively narrow absorption range within the solar spectrum, which inherently restricts the overall solar-energy conversion efficiency. To address this limitation, substantial effort has been focused on integrating synthetic light-harvesting materials with photosynthetic proteins to create biohybrid systems with broadened spectral absorption.

Two principal classes of synthetic light harvesters have proven particularly effective: organic fluorophores and semiconductor quantum dots (QDs). Early work by Gordiichuk et al.^244^ and more recently by Morlock et al.^139^ demonstrated covalent conjugation of organic fluorophores such as ATTO 590 and ATTO 532 to PSI. These fluorophores act as auxiliary antenna systems, harvesting light in spectral regions where PSI absorbs only weakly and transferring the excitation energy efficiently to the PSI core. This strategy effectively addresses the so-called “green gap” in PSI absorption.

A related supramolecular approach was applied to the photosynthetic bacterium *Rba. sphaeroides*, where an organic dye was used as a light-harvesting bridge between cyt *c* and a bRC, thereby enhancing overall photoconversion efficiency..^245^ Similarly, fluorophores including Alexa647, Alexa680, Alexa750, and ATTO647N were covalently attached to RC–LH1 complexes from *Rhodopseudomonas palustris.* ^246^ These modifications significantly improved both the quantum yield of charge separation and the overall photocurrent generation activity. Dewa et al. further demonstrated quantitative agreement between energy-transfer efficiencies in native antenna systems and their biohybrid analogues, confirming that synthetic antenna systems can function comparably to natural light-harvesting complexes.^247^

Semiconductor QDs have emerged as particularly versatile broadband antenna systems because their absorption and emission properties can be tuned precisely through particle size and composition. Liu et al. engineered bRCs from *Rba. sphaeroides* containing polyhistidine tags for tethering to water-soluble CdTe QDs.^248^ These studies provided important mechanistic insight into self-assembly processes and highly efficient excitation-energy transfer in QD–RC conjugates. The exceptional efficiency of these hybrid systems was later quantitatively confirmed by Amoruso et al., who demonstrated highly efficient excitation-energy transfer in quantum dot–bRC nanoconjugates.^249^

While synthetic light-harvesting materials can broaden the spectral response of photosynthetic proteins, they often suffer from limitations such as high cost, photobleaching of organic dyes, and the potential toxicity of semiconductor nanoparticles. To address these challenges, Liu et al. engineered a chimeric pigment–protein complex that integrates chlorophyll- and bacteriochlorophyll-based light-harvesting systems within a single biohybrid architecture, thereby enabling broad-spectrum solar-energy conversion using an entirely biological assembly (Figure 14).^250^ In a related strategy, Hartmann et al. combined cyanobacterial phycobilisomes with PSII in vitro to extend the absorption range of PSII-based biophotoelectrodes.^251^ Incorporation of these phycobilisome–PSII supercomplexes into Os-complex-modified hydrogels deposited on macroporous ITO electrodes resulted in a pronounced enhancement of the wavelength-dependent incident photon-to-electron conversion efficiency.

### 5.3 Overcoming Charge Recombination Processes

A central challenge in the development of efficient biophotoelectrochemical systems is the suppression of charge recombination pathways. In biohybrid photoelectrodes, photogenerated electrons can readily recombine with oxidized intermediates or follow nonproductive pathways, thereby limiting both photocurrent and photovoltage. Recent advances have therefore focused on engineering the biological–electrode interface to promote directional electron transfer and minimize recombination losses.

Noji et al. demonstrated that the structural integrity and native electrostatic environment of the RC–LH1 complex from *Rps. palustris* are critical for efficient photocurrent generation, highlighting the importance of preserving the native lipid membrane surrounding the photosynthetic complex.^252^ The retained lipid environment stabilizes the long-lived charge-separated state (P^+^Q_B_^–^), thereby reducing recombination and enhancing photocurrent generation.

A complementary strategy involves the passivation of recombination-active sites at the electrode interface. Olmos et al. investigated the biofunctionalization of *p*-doped silicon with cytochrome *c*_553_ and showed that the immobilized redox protein acts both as a molecular passivation layer and as an efficient electron mediator.^253^ This biointerface reduced the interfacial charge recombination and improved charge extraction in PSI-based all-solid-state devices.

Another promising approach relies on enforcing vectorial electron transport through the formation of highly ordered anisotropic monolayers of PSI. Zhao et al. fabricated uniformly oriented PSI monolayers on gold electrodes, enabling directional electron transfer from the donor side of PSI toward the acceptor side, thereby mimicking the vectorial charge separation of natural photosynthesis.^125^ This anisotropic arrangement suppresses isotropic electron transfer and minimizes short-circuiting pathways, enabling more efficient photoelectrochemical water splitting. Building upon this concept, Wang et al. developed mixed PSI monolayers with improved packing and interfacial connectivity, resulting in enhanced directional electron transport and reduced recombination losses^134^ Although the Langmuir–Blodgett approach provides excellent orientational control, its confinement to essentially monolayer architectures inherently limits protein loading and therefore the attainable photocurrent densities.

**Figure 14:**
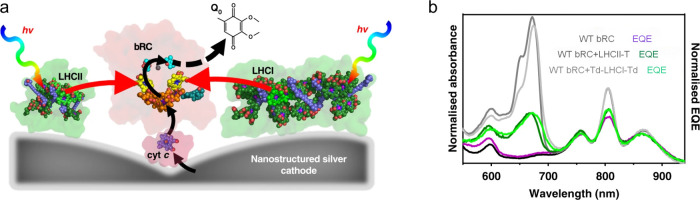
Biohybrid light-harvesting assemblies combining plant antenna complexes with purple bacterial reaction centers. (a) Schematic representation of photocurrent generation at a nanostructured silver electrode; black arrows indicate electron-transfer pathways, whereas red arrows denote excitation-energy transfer processes. (b) Comparison of solution absorbance spectra and wavelength-dependent external quantum efficiency (EQE) spectra for wild-type (WT) bRCs and hybrid assemblies containing WT bRCs coupled to either light-harvesting complex II (LHCII) or a light-harvesting complex I (LHCI) variant. Reproduced with the permission from ref 250. Copyright 2020 Springer Nature.

### 5.4 Prolonging Operational Lifetime

One of the most significant limitations of biophotoelectrochemical systems is the intrinsic instability of photosynthetic proteins under the electrochemical operating conditions. Photodamage, oxidative stress, and protein denaturation at interfaces often restrict operational lifetimes to only hours or days.^178^ Recent advances, however, have demonstrated strategies capable of extending functional stability from days to years.

A major degradation pathway arises from the formation of reactive oxygen species (ROS) generated during the oxygen reduction. Zhao et al. showed that operating a PSI-based photocathode under anaerobic conditions significantly prolonged its lifetime by suppressing ROS formation.^122^ In related work, PSI-based photocathodes employing quinones as electron acceptors enabled efficient photocurrent generation under anoxic conditions, thereby bypassing oxygen reduction and mitigating oxidative degradation.^124^ Szalkowski et al. further demonstrated that the photodamage dynamics of PSI strongly depend on the excitation wavelength and confirmed that anoxic conditions substantially improve photostability.^187^ In addition, they achieved an approximately 50-fold plasmonic enhancement of PSI fluorescence and improved photostability using planar metallic nanostructures, highlighting the potential of engineered nanointerfaces to simultaneously enhance performance and stability.

One of the most remarkable advances in long-term operational stability was reported by Friebe et al., who demonstrated that maintaining continuous electron transfer from the RC–LH1 complex of *Rba. sphaeroides* to an IO-mITO electrode dramatically improved photoelectrode durability.^170^ Under these conditions, the biohybrid electrode remained operational for 33 days and retained activity after storage for more than two years. Sustained electron flow prevents the accumulation of long-lived charge-separated states that otherwise promote photoinhibition and irreversible degradation.

## 6 Conclusion

The isolation of the photosystem protein complexes from their native, multicomponent photosynthetic electron transport chains and their rewiring into electrode architectures have led to remarkable performance in light energy conversion. In some cases, photosystem-based systems have achieved light-driven electron transfer rates and efficiencies that rival or even exceed those of natural photosynthesis while often relying on architectures far simpler than conventional photovoltaic devices. Over the past decade, the field has advanced substantially, achieving high photocurrent densities, fully integrated devices with high energy conversion efficiencies, and a deep mechanistic understanding of the processes governing performance losses. These advances have enabled proof-of-concept applications ranging from biophotovoltaic electricity generation to photoelectrochemical water-splitting for hydrogen production and CO_2_ reduction.

These successes rest on two main complementary development strategies. The first focuses on devices in which the photosystem itself serves as the sole photoactive component. The second incorporates photosystems into established photovoltaic or photoelectrode architectures to augment the device performance. The latter strategy has proven particularly successful for light-to-electricity conversion, where integration with semiconductor materials has yielded comparatively high power conversion efficiencies. In contrast, systems relying exclusively on photosystems as light absorbers have shown particular promise for solar-fuel generation, especially for light-driven hydrogen evolution.

Despite this progress, a notable disconnect remains between advances in biophotoelectrode development and their integration into complete biophotovoltaic devices. PSI photocathodes, for example, have been extensively studied as half-cells on metal- or carbon-based electrodes, yet in full devices, PSI is more commonly employed as a photoanode in combination with semiconductor materials. This disparity largely arises from the challenge of suppressing the charge recombination. Achieving high-performance photoelectrodes that rely exclusively on photosystems will therefore require strategies that effectively mitigate the recombination losses. Such solutions may draw inspiration from classical photoelectrode design principles while preserving the unique advantages of biological light-harvesting. Recent advances have also drawn inspiration from natural photosynthesis, replicating the compartmentalization of the various components of the electron-transfer chain leading to effective suppression of recombination.

More generally, the field has demonstrated several breakthroughs in which photosystem-based electrodes exhibit at least one of the key attributes among robustness, scalability, efficiency, or simplicity of fabrication that are required for applications. These advances are paving the way toward the development of biophotoelectrodes that fulfill all of these criteria, ultimately enabling their deployment alongside classical inorganic solar energy technologies.

## Supplementary Material



## Notes

Performances of biophotoelectrodes based on photosynthetic proteins (PDF)
