# Supplementary material for: Photosystem-Based Biophotoelectrodes for Solar Energy Conversion
Source: Chem Rev. 2026 Jul 21;126(15):8456–86. doi: 10.1021/acs.chemrev.6c00021 (PMC13474563; doi:10.1021/acs.chemrev.6c00021)
Supplement: Supplementary file 1 [file cr6c00021_si_001.pdf]

Supplementary Information for

Photosystem-based Biophotoelectrodes for Solar Energy Conversion

Huijie Zhang*[1] and Nicolas Plumeré*[2]

1 School of New Energy, Nanjing University of Science and Technology, Jiangyin, Jiangsu 214443, China.

2 Technical University of Munich, Campus Straubing for Biotechnology and Sustainability, Professorship for Electrobiotechnology, Uferstraße 53, 94315, Straubing, Germany.

Email: huijie.zhang@njust.edu.cn, nicolas.plumere@tum.de

This PDF file includes:

Table S1. Performances of biophotoelectrodes based on photosynthetic proteins.

Table S1. Performances of biophotoelectrodes based on photosynthetic proteins.

	Protein	Immobilization	Electrode	Type	ET	Donor/ Acceptor	j_{photo} μAcm^{-2}	E_{appied}	E_{onset}	IQE %	EQE %	stability	illumination	Year& Refs
**PSI**														
1	PS I spinach	physical adsorption	Au-SAM	cathode	DET	MV^{++}	-0.01	0.3 V vs NHE					cold light with red filter	2007[1]
2	PS I spinach	covalent binding	Au-SAM	cathode	MET	Asc/ DCPIP	-0.1	-0.1 V vs Ag/ AgCl						2008[2]
3	PS I spinach	electrodeposition	Au	cathode	MET	Asc/ DCPIP	-2					>280 days storage	white light 95 mW cm^{-2}	2010[3]
4	PSI *T. elongatus*	redox polymer	Au	cathode	MET	MV^{++}	-29	0 V vs Ag/ AgCl	0.2 V vs Ag/ AgCl				680 nm 1.8 mW cm^{-2}	2011[4]
5	PSI *T. elongatus*	physical adsorption	Au-SAM	cathode	MET	MV^{++}	-75 nA	-0.1 V vs Ag/ AgCl					> 676 nm 1.4 mW cm^{-2}	2013[5]
6	PSI *T. elongatus*	redox polymer	GCE	cathode	MET	MV^{++}	-322	0 V vs Ag/ AgCl	0.35 V vs Ag/ AgCl			$\tau_{1/2}$=15 h at 1 mW cm^{-2}	685 nm 40 mW cm^{-2}	2014[6]
7	PSI *T. elongatus*	physical adsorption	Graphene	cathode	DET	MV^{++}	-4.5	-0.22 V vs Ag/ AgCl					white light 100 mW cm^{-2}	2015[7]
8	PSI *T. elongatus*	covalent binding	graphene	cathode	DET	MV^{++}	-135	-0.56 V vs Ag/ AgCl					white light 100 mW cm^{-2}	2015[8]
9	PSI *Synechocystsis* sp. PCC 6803	Electrospray	TiO_2	anode	DET	Asc/ DCPIP	4150	0.3 V vs Ag/ AgCl					250−900 nm 220 mW cm^{-2}	2015[9]
10	PSI *T. elongatus*	protein film	Au-SAM	cathode	MET cyt c	MV^{++}	-24	−0.1 V vs Ag/ AgCl		24	4.1		white light 20 mW cm^{-2}	2016[10]

11	PSI *T. elongatus*	3D electrode	mesoITO	cathode	MET cyt c	O_2	-148	-0.1 V vs Ag/AgCl		39	11		white light 60 mW cm^{-2}	2016[11]
12	PSI *T. elongatus*	covalent binding	carbon nanotube	cathode	MET cyt c	MV^{++}	-18	-0.1 V vs. Ag/AgCl				90% photocurrent after 30 mins illumination	white light 100 mW cm^{-2}	2017[12]
13	PS I *Synechocystsis* sp. PCC 6803	physical adsorption	carbon nanotube	anode	DET	PMS	0.08							2017[13]
14	PSI *C. merolae*	physical adsorption	graphene	cathode	DET	O_2	-0.37	-0.3 V vs Ag/ AgCl					white light 100 mW cm^{-2}	2018[14]
15	PSI *T. elongatus*	redox polymer	Au	cathode	MET	MV^{++}	-11	0 V vs Ag/AgCl					white light 280 mW cm^{-2}	2018[15]
16	PSI *T. elongatus*	physical adsorption	Au-SAM fullerenes	cathode	DET	MV^{++}	-15	-0.3 V vs. Ag/AgCl	0.16 V vs Ag/AgCl	10		80% photocurrent after 30 mins illumination	white light 100 mW cm^{-2}	2019[16]
17	PSI *T. elongatus*	redox polymer	Au	cathode	MET	Q_0	-140	0.3 V vs SHE				> 15h	white light 80 mW cm^{-2}	2019[17]
18	PSI *T. elongatus*	Langmuir-Blodgett	Au	cathode	MET	MV^{++}	-4	0.21 V vs SHE					> 600 nm 43 mW cm^{-2}	2019[18]
19	PSI *T. elongatus*	3D electrode	mesoITO	cathode	MET cyt c	Q_0	-270	-0.15 V vs Ag/AgCl				>30 min	white light 100 mW cm^{-2}	2019[19]
20	PSI *T. elongatus*	covalent binding	pyrolytic graphite	anode	DET	Asc/ DCPIP	0.03	-0.15 V vs Ag/AgCl					680 nm 13.3 mW cm^{-2}	2020[20]
21	PSI spinach	3D electrode	MacroITO	cathode	MET	O_2	-42	OCP					white light 100 mW cm^{-2}	2020[21]
22	PSI *Synechocystsis* sp. PCC 6803	electrodeposi ton	FTO	cathode	MET Asc/D CPIP	Asc	-0.5	0.1 V vs SHE		0.37	0.02		685 nm 5.8 mW cm^{-2}	2020[22]
23	PSI *T. elongatus*	Airbrush spray-coating	porousITO	cathode	MET	MV^{++}	-30	0 V vs Ag/AgCl					> 685 nm 20 mW cm^{-2}	2020[23]

24	PSI *C. merolae*	Physical Adsorption	Graphene	cathode	DET	O_2	-0.774	-0.3 V vs Ag/ AgCl					white light 100 mW cm^{-2}	2021[24]
25	PSI *T. elongatus*	3D electrode	3D ITO	cathode	MET cyt c	O_2	-6	-0.2 V vs Ag/ AgCl			0.14		white light 80 mW cm^{-2}	2021[25]
26	PSI *T. elongatus*	3D electrode	3D rGO	cathode	MET cyt c	O_2	-14	-0.15 V vs Ag/AgCl.	0.07 V vs Ag/AgCl		0.07		white light 100 mW cm^{-2}	2021[26]
27	PSI *T. elongatus*	Langmuir-Blodgett	Au	cathode	MET	MV^{++}	-9	0.21 V vs SHE					red light 51 mW cm^{-2}	2021[27]
28	PSI spinach	conducting film	Au-SAM	cathode	MET	Q_0	-0.4						white light 100 mW cm^{-2}	2021[28]
29	PSI *T. elongatus*	3D electrode	3D ITO	cathode	DET	O_2	-10.1	-0.1V vs Ag/AgCl	0.4 V vs Ag/AgCl	4	0.8	90% photocurrent after 3 h illumination	white light 100 mW cm^{-2}	2022[29]
30	PSI *C. merolae*	electrodepost ion	FTO	cathode	MET Asc/D CPIP	Asc	-4.6	-0.18 V vs SHE		0.47			685 nm 5.8 mW cm^{-2}	2022[30]
31	PSI spinach	conducting film	carbon paper	cathode	DET	Q_0	-0.19	OCP					white light 100 mW cm^{-2}	2023[31]
32	PSI *T. vesticus*	3D electrode	3D ITO	cathode	DET	MV^{++}	-47	-0.1 V vs Ag/AgCl				$\tau_{1/2}$= 60 min	white light 100 mW cm^{-2}	2023[32]
33	PSI *T. elongatus*	Langmuir–Blodgett	Au	cathode	MET	MV^{++}	-4	0 V vs Ag/AgCl			0.01		red light 30 mW cm^{-2}	2023[33]
34	PSI *C. merolae*	3D electrode	silver island film	cathode	DET	O_2	-1.0	-0.3 V vs Ag/ AgCl					white light 100 mW cm^{-2}	2025[34]
35	PSI spinach	conducting film	ITO	cathode	MET	O_2	-30							2025[35]
36	PSI *T. elongatus*	redox polymer +SWCNTs	Au	cathode	MET	MV^{++}	-2000	0 V vs Ag/AgCl					white light 100 mW cm^{-2}	2025[36]
**PSII**														

37	PSII *S. elongatus*	physical adsorption	Au-SAM	anode	MET DQ	H_2O	0.108	0.62 V vs Ag/AgCl					650 nm 1.84 mW cm^{-2}	2005[37]
38	PSII *T. elongatus*	physical adsorption	Au-SAM	anode	MET DCBQ	H_2O	14	0.3 V vs Ag/AgCl					675 nm 1.77 mW cm^{-2}	2006[38]
39	PSII *T. elongatus*	redox polymer	Au	anode	MET	H_2O	45	0.3 V vs Ag/AgCl				$\tau_{1/2}$≈ 18 min	675 nm 2.65 mW cm^{-2}	2008[39]
40	PSII *T. elongatus*	3D electrode	mesoITO-SAM	anode	MET DCBQ	H_2O	4.5	0.5 V vs SHE				$\tau_{1/2}$ < 5 min	679 nm 10 mW cm^{-2}	2013[40]
41	PSII *T. elongatus*	3D electrode	IO-mesoITO	anode	MET DCBQ	H_2O	930	0.5 V vs NHE			17		679 nm 10 mW cm^{-2}	2015[41]
42	PSII spinach	conducting polymer	ITO	anode	MET BQ	H_2O	0.36	0.49 V vs NHE				$\tau_{1/2}$≈ 11 min	550 to 800 nm, P_{680nm} = 10 mW cm^{-2}	2016[42]
43	PSII *T. elongatus*	redox polymer+3D electrode	IO-mesoITO	anode	MET	H_2O	410	0.5 V vs SHE	0.3 V vs SHE		4.4	$\tau_{1/2}$≈ 4.3 min	685 nm 10 mW cm^{-2}	2016[43]
44	PSII *T. elongatus*	3D electrode	TiO_2/ITO	anode	MET	H_2O	12	0.644 V vs SHE					590 nm 17.4 mW cm^{-2}	2016[44]
45	PSII *T. elongatus*	covalent binding	Au-SAM Pt NPs	anode	DET	DPC	0.015	0.1 V vs Ag/AgCl					680 nm 29 mW cm^{-2}	2017[45]
46	PSII *T. elongatus*	redox polymer	Au	anode	MET	H_2O	3.18	0.550 V vs SHE					> 600 nm 76 mW cm^{-2}	2018[46]
47	PSII *T. elongatus*	redox polymer	Au	anode	MET	H_2O	9.5	0.4 V vs Ag/AgCl			0.04	$\tau_{1/2}$≈ 37 min	685 nm 50 mW cm^{-2}	2018[47]
48	PSII *T. elongatus*	3D electrode	IO-mesoITO	anode	MET DCBQ	H_2O	185	0.5 V vs. SHE			33.5		685 nm 1 mW cm^{-2}	2018[48]
49	PSII and PSI spinach	physical adsorption	carbon nanotube	anode	DET	H_2O	1.25	0.3 V vs Ag/AgCl					> 450 nm 78 mW cm^{-2}	2019[49]

50	PSII *T. elongatus*	3D electrode	IO-mesoITO	anode	MET DCBQ	H_2O	160	0.5 V vs SHE				$\tau_{1/2}\approx$ 4 min	685 nm 10 mW cm^{-2}	2019[50]
51	PSII *T. elongatus*	airbrush spray-coating	porousITO	anode	MET	H_2O	22.5	0.3 V vs Ag/AgCl					> 685 nm 20 mW cm^{-2}	2020[23]
52	PSII spinach	3D electrode	macroporous carbon electrode	anode	MET DCBQ	H_2O	18.1	OCP		1.20		10 h	white light 80 mW cm^{-2}	2021[51]
**RC/RC-LH1**														
53	bRC *Rba. sphaeroides*	conducting film	GCE	anode	DET	DT	5	-0.05 V vs SCE					>600 nm 10 mW cm^{-2}	2002[52]
54	bRC *Rba. sphaeroides*	physical adsorption	Au-SAM	anode	DET	DT	0.05						>600 nm 10 mW cm^{-2}	2002[53]
55	bRC *Rba. sphaeroides*	physical adsorption	Au-SAM	cathode	MET cyt c	Q_2	-0.16	-0.2 V vs Ag/AgCl					>700 nm 100 mW cm^{-2}	2006[54]
56	bRC *Rba. sphaeroides*	physical adsorption	Au-SAM	cathode	MET cyt c	Q_2	-0.16	0 V vs. NHE					>700 nm 100 mW cm^{-2}	2007[55]
57	RC-LH1 *Rs. rubrum*	physical adsorption	APS-ITO	cathode	DET	MV^{++}	-0.01	-0.2 V vs Ag/AgCl					880 nm	2007[56]
58	RC-LH1 *Rps. palustris*	physical adsorption	Au-SAM	cathode	DET	MV^{++}	-0.005	0.2 V vs Ag/AgCl					880 nm	2007[57]
59	memb *Rba. sphaeroides*	physical adsorption	Au	cathode	MET cyt c	Q_0	-10	−0.1 V vs SCE					885 nm 46 mW cm^{-2}	2010[58]
60	RC-LH1 *Rps. acidophila*	physical adsorption	Au	cathode	MET cyt c	Q_0	-25	−0.1 V vs SCE				10 h	870 nm 20 mW cm^{-2}	2011[59]
61	RC-LH1 *Rba. sphaeroides*	physical adsorption	Au-SAM	cathode	DET	MV^{++}	-0.005	-0.2 V vs Ag/AgCl					880 nm	2012[60]
62	bRC *Rba. sphaeroides*	physical adsorption	Au-SAM	cathode	MET cyt c	Q_2	-0.5	0.2 V vs SHE					white light 80 mW cm^{-2}	2014[61]

63	RC-LH1 *Rps. acidophila*	Langmuir–Blodgett	Au	cathode	MET cyt c	Q_0	-45	−0.1 V vs SCE		32	0.28		880 nm 23 mW cm^{-2}	2014[62]
64	RC-LH1 *Rba. sphaeroides*	physical adsorption	Au	cathode	DET	Cp_2Fe	-2	OCP			0.21		white light 80 mW cm^{-2}	2015[63]
65	RC-LH1 *Rba. sphaeroides*	3D electrode	rough Ag	cathode	MET cyt c	Q_0	-166 -416 (4 sun)	-0.05 V vs Ag/AgCl	0.35 vs SHE	39	0.13	$\tau_{1/2}$=8 h	white light 100 mW cm^{-2}	2016[64]
66	bRC *Rba. sphaeroides*	physical adsorption	Au-SAM	cathode	MET cyt c	Q_0	-12	−0.1 V vs SCE					870 nm 45 mW cm^{-2}	2016[65]
67	bRC *Rba. sphaeroides*	3D electrode	ATO	cathode	MET cyt c	Q_0	-2	OCP					800 nm 5 mW cm^{-2}	2016[66]
68	bRC *Rba. sphaeroides*	3D electrode	rough Ag-SAM	cathode	MET cyt c	Q_0	-103	-0.05 V vs Ag/AgCl		13	0.31		870 nm 46 mW cm^{-2}	2017[67]
69	bRC *Rba. sphaeroides*	physical adsorption	graphene	cathode	MET AmMe Fc	Q_0	-1.2	-0.1V vs Ag/ AgCl				18 h	870 nm 9 mW cm^{-2}	2018[68]
70	bRC *Rba. sphaeroides*	redox polymer	FTO	cathode	MET	Q_0	-52	+360 mV vs SHE		50			860 nm 29 mW cm^{-2}	2020[69]
71	bRC *Rba. sphaeroides*	covalent binding	Au	anode	DET	QH_2	12	OCP					865 nm 70 mW cm^{-2}	2021[70]
72	bRC *Rba. sphaeroides*	3D electrode	IO-mesoITO	cathode	MET cyt c	Q_0	-4600	0.16 V vs SHE	0.3 vs SHE	7	4.70	$\tau_{1/2}$=20 h	870 nm 42 mW cm^{-2}	2022[71]

References:

(1) Ciobanu, M.; Kincaid, H. A.; Lo, V.; Dukes, A. D.; Kane Jennings, G.; Cliffel, D. E. Electrochemistry and photoelectrochemistry of photosystem I adsorbed on hydroxyl-terminated monolayers. *J. Electroanal. Chem.* **2007**, *599* (1), 72-78. DOI: 10.1016/j.jelechem.2006.09.019.

(2) Faulkner, C. J.; Lees, S.; Ciesielski, P. N.; Cliffel, D. E.; Jennings, G. K. Rapid assembly of photosystem I monolayers on gold electrodes. *Langmuir* **2008**, *24* (16), 8409-8412. DOI: 10.1021/la800670b.

(3) Ciesielski, P. N.; Hijazi, F. M.; Scott, A. M.; Faulkner, C. J.; Beard, L.; Emmett, K.; Rosenthal, S. J.; Cliffel, D.; Kane Jennings, G. Photosystem I – Based biohybrid photoelectrochemical cells. *Bioresour. Technol.* **2010**, *101* (9), 3047-3053. DOI: 10.1016/j.biortech.2009.12.045.

(4) Badura, A.; Guschin, D.; Kothe, T.; Kopczak, M. J.; Schuhmann, W.; Rögner, M. Photocurrent generation by photosystem 1 integrated in crosslinked redox hydrogels. *Energy Environ. Sci.* **2011**, *4* (7), 2435-2440, 10.1039/C1EE01126J. DOI: 10.1039/C1EE01126J.

(5) Manocchi, A. K.; Baker, D. R.; Pendley, S. S.; Nguyen, K.; Hurley, M. M.; Bruce, B. D.; Sumner, J. J.; Lundgren, C. A. Photocurrent generation from surface assembled photosystem I on alkanethiol modified electrodes. *Langmuir* **2013**, *29* (7), 2412-2419. DOI: 10.1021/la304477u.

(6) Kothe, T.; Pöller, S.; Zhao, F.; Fortgang, P.; Rögner, M.; Schuhmann, W.; Plumeré, N. Engineered electron-transfer chain in photosystem 1 based photocathodes outperforms electron-transfer rates in natural photosynthesis. *Chem. Eur. J.* **2014**, *20* (35), 11029-11034. DOI: 10.1002/chem.201402585.

(7) Feifel, S. C.; Lokstein, H.; Hejazi, M.; Zouni, A.; Lisdat, F. Unidirectional photocurrent of photosystem I on π-system-modified graphene electrodes: nanobionic approaches for the construction of photobiohybrid systems. *Langmuir* **2015**, *31* (38), 10590-10598. DOI: 10.1021/acs.langmuir.5b01625.

(8) Feifel, S. C.; Stieger, K. R.; Lokstein, H.; Lux, H.; Lisdat, F. High photocurrent generation by photosystem I on artificial interfaces composed of π-system-modified graphene. *J. Mater. Chem. A* **2015**, *3* (23), 12188-12196, 10.1039/C5TA00656B. DOI: 10.1039/C5TA00656B.

(9) Shah, V. B.; Henson, W. R.; Chadha, T. S.; Lakin, G.; Liu, H.; Blankenship, R. E.; Biswas, P. Linker-free deposition and adhesion of photosystem I onto nanostructured

TiO_2 for biohybrid photoelectrochemical cells. *Langmuir* **2015**, *31* (5), 1675-1682. DOI: 10.1021/la503776b.

(10) Stieger, K. R.; Ciornii, D.; Kölsch, A.; Hejazi, M.; Lokstein, H.; Feifel, S. C.; Zouni, A.; Lisdat, F. Engineering of supramolecular photoactive protein architectures: the defined co-assembly of photosystem I and cytochrome c using a nanoscaled DNA-matrix. *Nanoscale* **2016**, *8* (20), 10695-10705, 10.1039/C6NR00097E. DOI: 10.1039/C6NR00097E.

(11) Stieger, K.; Feifel, S.; Lokstein, H.; Hejazi, M.; Zouni, A.; Lisdat, F. Biohybrid architectures for efficient light-to-current conversion based on photosystem I within scalable 3D mesoporous electrodes. *J. Mater. Chem. A* **2016**, *4* (43), 17009-17017. DOI: 10.1039/C6TA07141D.

(12) Ciornii, D.; Feifel, S. C.; Hejazi, M.; Kölsch, A.; Lokstein, H.; Zouni, A.; Lisdat, F. Construction of photobiocathodes using multi-walled carbon nanotubes and photosystem I. *Phys Status Solidi A* **2017**, *214* (9), 1700017. DOI: 10.1002/pssa.201700017.

(13) Nii, D.; Miyachi, M.; Shimada, Y.; Nozawa, Y.; Ito, M.; Homma, Y.; Ikehira, S.; Yamanoi, Y.; Nishihara, H.; Tomo, T. Conjugates between photosystem I and a carbon nanotube for a photoresponse device. *Photosynth. Res.* **2017**, *133* (1), 155-162. DOI: 10.1007/s11120-016-0324-0.

(14) Kiliszek, M.; Harputlu, E.; Szalkowski, M.; Kowalska, D.; Unlu, C. G.; Haniewicz, P.; Abram, M.; Wiwatowski, K.; Niedziółka-Jönsson, J.; Maćkowski, S.; et al. Orientation of photosystem I on graphene through cytochrome c553 leads to improvement in photocurrent generation. *J. Mater. Chem. A* **2018**, *6* (38), 18615-18626, 10.1039/C8TA02420K. DOI: 10.1039/C8TA02420K.

(15) Zhao, F.; Hardt, S.; Hartmann, V.; Zhang, H.; Nowaczyk, M. M.; Rögner, M.; Plumeré, N.; Schuhmann, W.; Conzuelo, F. Light-induced formation of partially reduced oxygen species limits the lifetime of photosystem 1-based biocathodes. *Nat. Commun.* **2018**, *9* (1), 1973. DOI: 10.1038/s41467-018-04433-z.

(16) Ciornii, D.; Kölsch, A.; Zouni, A.; Lisdat, F. Exploiting new ways for a more efficient orientation and wiring of PSI to electrodes: A fullerene C70 approach. *Electrochim. Acta* **2019**, *299*, 531-539. DOI: 10.1016/j.electacta.2019.01.032.

(17) Zhao, F.; Ruff, A.; Rögner, M.; Schuhmann, W.; Conzuelo, F. Extended operational lifetime of a photosystem-based bioelectrode. *J. Am. Chem. Soc* **2019**, *141* (13), 5102-5106. DOI: 10.1021/jacs.8b13869.

(18) Zhao, F.; Wang, P.; Ruff, A.; Hartmann, V.; Zacarias, S.; Pereira, I. A. C.; Nowaczyk, M. M.; Rögner, M.; Conzuelo, F.; Schuhmann, W. A photosystem I monolayer with anisotropic electron flow enables Z-scheme like photosynthetic water splitting. *Energy Environ. Sci.* **2019**, *12* (10), 3133-3143, 10.1039/C9EE01901D. DOI: 10.1039/C9EE01901D.

(19) Ciornii, D.; Kölsch, A.; Zouni, A.; Lisdat, F. A precursor-approach in constructing 3D ITO electrodes for the improved performance of photosystem I-cyt c photobioelectrodes. *Nanoscale* **2019**, *11* (34), 15862-15870, 10.1039/C9NR04344F. DOI: 10.1039/C9NR04344F.

(20) Zhu, W.; Salles, R.; Miyachi, M.; Yamanoi, Y.; Tomo, T.; Takahashi, H.; Nishihara, H. Photoelectric conversion system composed of gene-recombined photosystem I and platinum nanoparticle nanosheet. *Langmuir* **2020**, *36* (23), 6429-6435. DOI: 10.1021/acs.langmuir.0c00647.

(21) Wolfe, K. D.; Dervishogullari, D.; Stachurski, C. D.; Passantino, J. M.; Kane Jennings, G.; Cliffel, D. E. Photosystem I Multilayers within Porous Indium Tin Oxide Cathodes Enhance Mediated Electron Transfer. *ChemElectroChem* **2020**, *7* (3), 596-603. DOI: 10.1002/celc.201901628.

(22) Szewczyk, S.; Białek, R.; Burdziński, G.; Gibasiewicz, K. Photovoltaic activity of electrodes based on intact photosystem I electrodeposited on bare conducting glass. *Photosynth. Res* **2020**, *144* (1), 1-12. DOI: 10.1007/s11120-020-00722-1.

(23) Bobrowski, T.; Conzuelo, F.; Ruff, A.; Hartmann, V.; Frank, A.; Erichsen, T.; Nowaczyk, M. M.; Schuhmann, W. Scalable fabrication of biophotoelectrodes by means of automated airbrush spray-coating. *ChemPlusChem* **2020**, *85* (7), 1396-1400. DOI: 10.1002/cplu.202000291.

(24) Izzo, M.; Jacquet, M.; Fujiwara, T.; Harputlu, E.; Mazur, R.; Wróbel, P.; Góral, T.; Unlu, C. G.; Ocakoglu, K.; Miyagishima, S.; et al. Development of a novel nanoarchitecture of the robust photosystem I from a volcanic microalga cyanidioschyzon merolae on single layer graphene for improved photocurrent generation. In *Int. J. Mol. Sci.*, 2021; Vol. 22.

(25) Nioradze, N.; Ciornii, D.; Kölsch, A.; Göbel, G.; Khoshtariya, D. E.; Zouni, A.; Lisdat, F. Electrospinning for building 3D structured photoactive biohybrid electrodes. *Bioelectrochemistry* **2021**, *142*, 107945. DOI: 10.1016/j.bioelechem.2021.107945.

(26) Morlock, S.; Subramanian, S. K.; Zouni, A.; Lisdat, F. Scalable three-dimensional photobioelectrodes made of reduced graphene oxide combined with photosystem I. *ACS Appl. Mater. Interfaces* **2021**, *13* (9), 11237-11246. DOI: 10.1021/acsami.1c01142.

(27) Wang, P.; Frank, A.; Zhao, F.; Szczesny, J.; Junqueira, J. R. C.; Zacarias, S.; Ruff, A.; Nowaczyk, M. M.; Pereira, I. A. C.; Rögner, M.; et al. Closing the gap for electronic short-circuiting: photosystem I mixed monolayers enable improved anisotropic electron flow in biophotovoltaic devices. *Angew. Chem., Int. Ed.* **2021**, *60* (4), 2000-2006. DOI: 10.1002/anie.202008958.

(28) Wolfe, K. D.; Gargye, A.; Mwambutsa, F.; Than, L.; Cliffel, D. E.; Jennings, G. K. Layer-by-Layer assembly of photosystem I and PEDOT:PSS biohybrid films for photocurrent generation. *Langmuir* **2021**, *37* (35), 10481-10489. DOI: 10.1021/acs.langmuir.1c01385.

(29) Morlock, S.; Subramanian, S. K.; Zouni, A.; Lisdat, F. Bio-inorganic hybrid structures for direct electron transfer to photosystem I in photobioelectrodes. *Biosens. Bioelectron.* **2022**, *214*, 114495. DOI: 10.1016/j.bios.2022.114495.

(30) Szewczyk, S.; Goyal, A.; Abram, M.; Burdziński, G.; Kargul, J.; Gibasiewicz, K. Electron transfer in a bio-photoelectrode based on photosystem I multilayer immobilized on the conducting glass. In *Int. J. Mol. Sci.*, 2022; Vol. 23.

(31) Stachurski, C. D.; Williams, J. M.; Tabaquin, P. M.; Wood, E. D.; Phambu, N.; Jennings, G. K.; Cliffel, D. E. Utilizing toray paper as a metal-Free, high Surface area electrode for photosystem I–driven mediated electron transfer. *Energy Technol.* **2023**, *11* (4), 2201077. DOI: 10.1002/ente.202201077.

(32) Morlock, S.; Subramanian, S. K.; Zouni, A.; Lisdat, F. Closing the green gap of photosystem I with synthetic fluorophores for enhanced photocurrent generation in photobiocathodes. *Chem. Sci.* **2023**, *14* (7), 1696-1708, 10.1039/D2SC05324A. DOI: 10.1039/D2SC05324A.

(33) Wang, P.; Frank, A.; Zhao, F.; Nowaczyk, M. M.; Conzuelo, F.; Schuhmann, W. A biomimetic assembly of folded photosystem I monolayers for an improved light utilization in biophotovoltaic devices. *Bioelectrochemistry* **2023**, *149*, 108288. DOI: 10.1016/j.bioelechem.2022.108288.

(34) Szalkowski, M.; Kiliszek, M.; Harputlu, E.; Izzo, M.; Gokhan Unlu, C.; Mackowski, S.; Ocakoglu, K.; Kargul, J.; Kowalska, D. Bimodal functionality of highly conductive nanostructured silver film towards improved performance of photosystem I-based graphene photocathode. *Bioelectrochemistry* **2025**, *161*, 108825. DOI: 10.1016/j.bioelechem.2024.108825.

(35) Lowery, W. R.; Jennings, G. K.; Cliffel, D. E. Counteranion effects on the incorporation of photosystem I with poly(3,4-ethylenedioxythiophene) (PEDOT). *ACS Omega* **2025**, *10* (10), 10199-10204. DOI: 10.1021/acsomega.4c09448.

(36) Yu, Y.; Su, X.; Xing, T.; Zhao, X.; Zhang, Z.; Zhang, W.; Wang, X.; Zhao, W.; Li, M.; Zhao, F. Enhanced photocurrent generation of a bio-photocathode based on photosystem I integrated in solvated redox polymers films nanostructured by SWCNTs. *Bioelectrochemistry* **2025**, *165*, 108979. DOI: 10.1016/j.bioelechem.2025.108979.

(37) Maly, J.; Krejci, J.; Ilie, M.; Jakubka, L.; Masojídek, J.; Pilloton, R.; Sameh, K.; Steffan, P.; Stryhal, Z.; Sugiura, M. Monolayers of photosystem II on gold electrodes with enhanced sensor response—effect of porosity and protein layer arrangement. *Anal. Bioanal. Chem.* **2005**, *381* (8), 1558-1567. DOI: 10.1007/s00216-005-3149-9.

(38) Badura, A.; Esper, B.; Ataka, K.; Grunwald, C.; Wöll, C.; Kuhlmann, J.; Heberle, J.; Rögner, M. Light-driven water splitting for (Bio-)hydrogen production: photosystem 2 as the central part of a bioelectrochemical device. *Photochem. Photobiol.* **2006**, *82* (5), 1385-1390. DOI: 10.1562/2006-07-14-RC-969.

(39) Badura, A.; Guschin, D.; Esper, B.; Kothe, T.; Neugebauer, S.; Schuhmann, W.; Rögner, M. Photo-induced electron transfer between photosystem 2 via cross-linked redox hydrogels. *Electroanalysis* **2008**, *20* (10), 1043-1047. DOI: 10.1002/elan.200804191.

(40) Kato, M.; Cardona, T.; Rutherford, A. W.; Reisner, E. Covalent Immobilization of Oriented Photosystem II on a Nanostructured Electrode for Solar Water Oxidation. *J. Am. Chem. Soc* **2013**, *135* (29), 10610-10613. DOI: 10.1021/ja404699h.

(41) Mersch, D.; Lee, C.-Y.; Zhang, J. Z.; Brinkert, K.; Fontecilla-Camps, J. C.; Rutherford, A. W.; Reisner, E. Wiring of photosystem II to hydrogenase for photoelectrochemical water splitting. *J. Am. Chem. Soc* **2015**, *137* (26), 8541-8549. DOI: 10.1021/jacs.5b03737.

(42) Li, G.; Feng, X.; Fei, J.; Cai, P.; Li, J.; Huang, J.; Li, J. Interfacial assembly of photosystem II with conducting polymer films toward enhanced photo-bioelectrochemical cells. *Adv. Mater. Interfaces* **2017**, *4* (1), 1600619. DOI: 10.1002/admi.201600619.

(43) Sokol, K. P.; Mersch, D.; Hartmann, V.; Zhang, J. Z.; Nowaczyk, M. M.; Rögner, M.; Ruff, A.; Schuhmann, W.; Plumeré, N.; Reisner, E. Rational wiring of photosystem II to hierarchical indium tin oxide electrodes using redox polymers. *Energy Environ. Sci.* **2016**, *9* (12), 3698-3709, 10.1039/C6EE01363E. DOI: 10.1039/C6EE01363E.

(44) Brinkert, K.; Le Formal, F.; Li, X.; Durrant, J.; Rutherford, A. W.; Fantuzzi, A. Photocurrents from photosystem II in a metal oxide hybrid system: electron transfer pathways. *BBA - Bioenergetics* **2016**, *1857* (9), 1497-1505. DOI: 10.1016/j.bbabio.2016.03.004.

(45) Miyachi, M.; Ikehira, S.; Nishiori, D.; Yamanoi, Y.; Yamada, M.; Iwai, M.; Tomo, T.; Allakhverdiev, S. I.; Nishihara, H. Photocurrent generation of reconstituted photosystem II on a self-assembled gold film. *Langmuir* **2017**, *33* (6), 1351-1358. DOI: 10.1021/acs.langmuir.6b03499.

(46) Zhao, F.; Hartmann, V.; Ruff, A.; Nowaczyk, M. M.; Rögner, M.; Schuhmann, W.; Conzuelo, F. Unravelling electron transfer processes at photosystem 2 embedded in an Os-complex modified redox polymer. *Electrochim. Acta* **2018**, *290*, 451-456. DOI: 10.1016/j.electacta.2018.09.093.

(47) Hartmann, V.; Ruff, A.; Schuhmann, W.; Rögner, M.; Nowaczyk, M. M. Analysis of photosystem II electron transfer with natural PsbA-variants by redox polymer/protein biophotoelectrochemistry. *Photosynthetica* **2018**, *56* (1), 229-235. DOI: 10.1007/s11099-018-0775-y.

(48) Zhang, J. Z.; Bombelli, P.; Sokol, K. P.; Fantuzzi, A.; Rutherford, A. W.; Howe, C. J.; Reisner, E. Photoelectrochemistry of Photosystem II in Vitro vs in Vivo. *J. Am. Chem. Soc.* **2018**, *140* (1), 6-9. DOI: 10.1021/jacs.7b08563.

(49) Kim, I.; Jo, N.; Yang, M. Y.; Kim, J.; Jun, H.; Lee, G. Y.; Shin, T.; Kim, S. O.; Nam, Y. S. Directed nanoscale self-assembly of natural photosystems on nitrogen-doped carbon nanotubes for solar-energy harvesting. *ACS Appl. Bio Mater.* **2019**, *2* (5), 2109-2115. DOI: 10.1021/acsabm.9b00120.

(50) Fang, X.; Sokol, K. P.; Heidary, N.; Kandiel, T. A.; Zhang, J. Z.; Reisner, E. Structure–activity relationships of hierarchical three-dimensional electrodes with photosystem II for semiartificial photosynthesis. *Nano Lett.* **2019**, *19* (3), 1844-1850. DOI: 10.1021/acs.nanolett.8b04935.

(51) Tian, W.; Zhang, H.; Sibbons, J.; Sun, H.; Wang, H.; Wang, S. Photoelectrochemical water oxidation and longevous photoelectric conversion by a photosystem II electrode. *Adv. Energy Mater.* **2021**, *11* (30), 2100911. DOI: 10.1002/aenm.202100911.

(52) Zhao, J.; Ma, N.; Liu, B.; Zhou, Y.; Xu, C.; Kong, J. Photoelectrochemistry of photosynthetic reaction centers embedded in Al2O3 gel. *J. Photochem. Photobiol., A* **2002**, *152* (1), 53-60. DOI: 10.1016/S1010-6030(02)00188-0.

(53) Zhao, J.; Zou, Y.; Liu, B.; Xu, C.; Kong, J. Differentiating the orientations of photosynthetic reaction centers on Au electrodes linked by different bifunctional reagents. *Biosens. Bioelectron.* **2002**, *17* (8), 711-718. DOI: 10.1016/S0956-5663(02)00026-X.

(54) Lebedev, N.; Trammell, S. A.; Spano, A.; Lukashev, E.; Griva, I.; Schnur, J.

Conductive wiring of immobilized photosynthetic reaction center to electrode by cytochrome c. *J. Am. Chem. Soc* **2006**, *128* (37), 12044-12045. DOI: 10.1021/ja063367y.

(55) Trammell, S. A.; Griva, I.; Spano, A.; Tsoi, S.; Tender, L. M.; Schnur, J.; Lebedev, N. Effects of distance and driving force on photoinduced electron transfer between photosynthetic reaction centers and gold electrodes. *J. Phys. Chem. C* **2007**, *111* (45), 17122-17130. DOI: 10.1021/jp0740402.

(56) Suemori, Y.; Fujii, K.; Ogawa, M.; Nakamura, Y.; Shinohara, K.; Nakagawa, K.; Nagata, M.; Iida, K.; Dewa, T.; Yamashita, K.; et al. Molecular assembly of artificial photosynthetic antenna core complex on an amino-terminated ITO electrode. *Colloids Surf., B* **2007**, *56* (1), 182-187. DOI: 10.1016/j.colsurfb.2006.10.029.

(57) Kondo, M.; Nakamura, Y.; Fujii, K.; Nagata, M.; Suemori, Y.; Dewa, T.; Iida, K.; Gardiner, A. T.; Cogdell, R. J.; Nango, M. Self-assembled monolayer of light-harvesting core complexes from photosynthetic bacteria on a gold electrode modified with alkanethiols. *Biomacromolecules* **2007**, *8* (8), 2457-2463. DOI: 10.1021/bm070352z.

(58) Magis, G. J.; den Hollander, M.-J.; Onderwaater, W. G.; Olsen, J. D.; Hunter, C. N.; Aartsma, T. J.; Frese, R. N. Light harvesting, energy transfer and electron cycling of a native photosynthetic membrane adsorbed onto a gold surface. *BBA - Biomembranes* **2010**, *1798* (3), 637-645. DOI: 10.1016/j.bbamem.2009.12.018.

(59) den Hollander, M.-J.; Magis, J. G.; Fuchsenberger, P.; Aartsma, T. J.; Jones, M. R.; Frese, R. N. Enhanced photocurrent generation by photosynthetic bacterial reaction centers through molecular relays, light-Harvesting complexes, and direct protein–gold interactions. *Langmuir* **2011**, *27* (16), 10282-10294. DOI: 10.1021/la2013528.

(60) Kondo, M.; Iida, K.; Dewa, T.; Tanaka, H.; Ogawa, T.; Nagashima, S.; Nagashima, K. V. P.; Shimada, K.; Hashimoto, H.; Gardiner, A. T.; et al. Photocurrent and electronic activities of oriented-His-Tagged photosynthetic light-harvesting/reaction center core complexes assembled onto a gold electrode. *Biomacromolecules* **2012**, *13* (2), 432-438. DOI: 10.1021/bm201457s.

(61) Yaghoubi, H.; Li, Z.; Jun, D.; Lafalce, E.; Jiang, X.; Schlaf, R.; Beatty, J. T.; Takshi, A. Hybrid wiring of the rhodobacter sphaeroides reaction center for applications in bio-photoelectrochemical solar cells. *J. Phys. Chem. C* **2014**, *118* (41), 23509-23518. DOI: 10.1021/jp507065u.

(62) Kamran, M.; Delgado, J. D.; Friebe, V.; Aartsma, T. J.; Frese, R. N. Photosynthetic protein complexes as bio-photovoltaic building blocks retaining a high internal quantum efficiency. *Biomacromolecules* **2014**, *15* (8), 2833-2838. DOI: 10.1021/bm500585s.

(63) Yaghoubi, H.; Lafalce, E.; Jun, D.; Jiang, X.; Beatty, J. T.; Takshi, A. Large photocurrent response and external quantum efficiency in biophotoelectrochemical cells incorporating reaction center plus light harvesting complexes. *Biomacromolecules* **2015**, *16* (4), 1112-1118. DOI: 10.1021/bm501772x.

(64) Friebe, V. M.; Delgado, J. D.; Swainsbury, D. J. K.; Gruber, J. M.; Chanaewa, A.; van Grondelle, R.; von Hauff, E.; Millo, D.; Jones, M. R.; Frese, R. N. Plasmon-enhanced photocurrent of photosynthetic pigment proteins on nanoporous silver. *Adv. Funct. Mater.* **2016**, *26* (2), 285-292. DOI: 10.1002/adfm.201504020.

(65) Friebe, V. M.; Swainsbury, D. J. K.; Fyfe, P. K.; van der Heijden, W.; Jones, M. R.; Frese, R. N. On the mechanism of ubiquinone mediated photocurrent generation by a reaction center based photocathode. *BBA - Bioenergetics* **2016**, *1857* (12), 1925-1934. DOI: 10.1016/j.bbabio.2016.09.011.

(66) Carey, A.-M.; Zhang, H.; Mieritz, D.; Volosin, A.; Gardiner, A. T.; Cogdell, R. J.; Yan, H.; Seo, D.-K.; Lin, S.; Woodbury, N. W. Photocurrent generation by photosynthetic purple bacterial reaction centers interfaced with a porous antimony-doped tin oxide (ATO) electrode. *ACS Appl. Mater. Interfaces* **2016**, *8* (38), 25104-25110. DOI: 10.1021/acsami.6b07940.

(67) Friebe, V. M.; Millo, D.; Swainsbury, D. J. K.; Jones, M. R.; Frese, R. N. Cytochrome c provides an electron-funneling antenna for efficient photocurrent generation in a reaction center biophotocathode. *ACS Appl. Mater. Interfaces* **2017**, *9* (28), 23379-23388. DOI: 10.1021/acsami.7b03278.

(68) Csiki, R.; Drieschner, S.; Lyuleeva, A.; Cattani-Scholz, A.; Stutzmann, M.; Garrido, J. A. Photocurrent generation of biohybrid systems based on bacterial reaction centers and graphene electrodes. *Diamond Relat. Mater.* **2018**, *89*, 286-292. DOI: 10.1016/j.diamond.2018.09.005.

(69) Białek, R.; Friebe, V.; Ruff, A.; Jones, M. R.; Frese, R.; Gibasiewicz, K. In situ spectroelectrochemical investigation of a biophotoelectrode based on photoreaction centers embedded in a redox hydrogel. *Electrochim. Acta* **2020**, *330*, 135190. DOI: 10.1016/j.electacta.2019.135190.

(70) Jun, D.; Zhang, S.; Grzędowski, A. J.; Mahey, A.; Beatty, J. T.; Bizzotto, D. Correlating structural assemblies of photosynthetic reaction centers on a gold electrode and the photocurrent - potential response. *iScience* **2021**, *24* (5), 102500. DOI: 10.1016/j.isci.2021.102500.

(71) Friebe, V. M.; Barszcz, A. J.; Jones, M. R.; Frese, R. N. Sustaining electron transfer pathways extends biohybrid photoelectrode stability to years. *Angew. Chem., Int. Ed.*

2022, *61* (24), e202201148. DOI: 10.1002/anie.202201148.
